# Probing the pH- and Voltage-Driven Intramolecular Folding Dynamics of the *c*‑MycC52 Promoter *i*‑Motif at the Single-Molecule Level

**DOI:** 10.1021/acs.analchem.6c04361

**Published:** 2026-09-17

**Authors:** Adina Cimpanu, Jonggwan Park, Yoonkyung Park, Loredana Mereuta, Tudor Luchian

**Affiliations:** † Department of Physics, Alexandru I. Cuza University, Iasi 700506, Romania; ‡ Department of Biomedical Science and Institute for Peptide Drugs (IPD), Chosun University, Gwangju 61452, Republic of Korea

## Abstract

The human *c*-Myc proto-oncogene is a transcription factor that regulates protein expression and is controlled by GC-rich regulatory sequences capable of adopting non-canonical DNA secondary structures. Here, we use the α-hemolysin (α-HL) protein nanopore to probe pH- and voltage-dependent effects on the stability of the *i*-motif formed by the 52-nucleotide cytosine-rich *c*-Myc promoter (*c*-MycC52). Under acidic conditions, electrophoretically driven *c*-MycC52 *i*-motifs are transiently trapped within the α-HL vestibule, generating pH-dependent electrical current signatures that are volumetrically and kinetically distinct and resolvable at the single-molecule level. The larger molecular volume measured in the acidic pH range, compared with that of linearized DNA at neutral pH, is consistent with the formation of an *i*-motif. In contrast, the applied transmembrane voltage does not induce detectable changes in the topology of the captured *i*-motif fragments. Kinetic modeling of current fluctuations within a continuous-time Markov chain framework reveals a subtle coupling between the acidic pH-dependent compaction topology of the captured *i*-motif and its local interactions with the α-HL vestibule interior and constriction zone. Although molar KCl concentrations (1 and 3 M) yield *c*-MycC52 *i*-motif structures of comparably similar volumes at low pH, reduced charge screening at lower ionic strength enhances electrostatic repulsion along the negatively charged DNA backbone, rendering the vestibule-captured folded structure kinetically more stable and more effective at occluding the α-HL constriction. These findings establish α-HL electrophysiology as a sensitive platform for distinguishing subtle differences in *i*-motif conformational stability, with potential applications in DNA secondary structure dynamics and disease-targeted diagnostic strategies.

## Graphical abstract

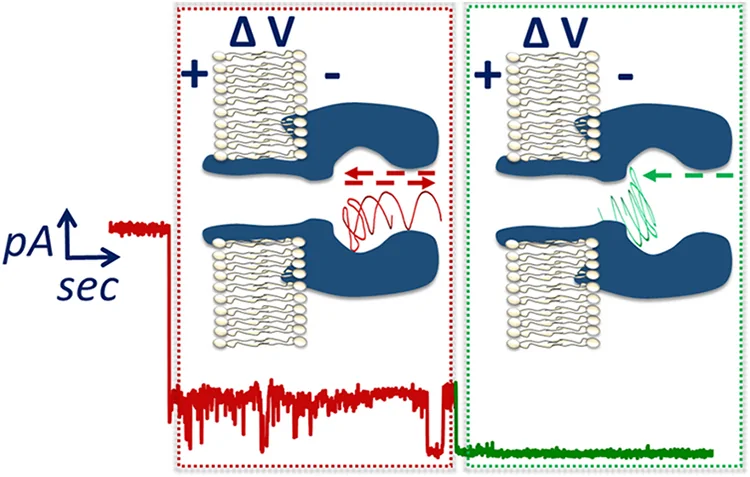

## Introduction

Structural studies of nucleic acids have undergone a significant conceptual expansion since the original X-ray diffraction elucidation of the DNA double helix^1^ and the subsequent characterization of the two right-handed helical forms, A-DNA and B-DNA,^2^ which serve as the canonical carriers of genetic information. This expanding structural landscape now encompasses a broad repertoire of non-canonical DNA conformations with established regulatory roles, including triplex DNA, hairpin and cruciform structures, G-quadruplexes (G4s), and *i*-motifs.^3^

The *i*-motif structure was first characterized for the hexanucleotide sequence d­(TCCCCC) under acidic conditions,^4^ revealing an architecture comprising two parallel-stranded duplexes intercalated in antiparallel orientation and stabilized by hemiprotonated cytosine–cytosine^+^ (C:C^+^) base pairs.^5^ It is now well established that *i*-motifs can form either intermolecularly—through the association of two (dimeric) or four (tetrameric) separate DNA strands—or intramolecularly, where four cytosine-rich tracts within a single strand fold into a monomeric *i*-motif topology.^6^

The C:C^+^ base pair is the defining stabilizing interaction of the *i*-motif, formed mostly under acidic conditions that permit cytosine N3 hemiprotonation. Each C:C^+^ pair contributes three hydrogen bonds, conferring high thermodynamic stability to the folded structure.^7^

Computational studies have established that the base-pairing energy of the C:C^+^ base pair (169.7 kJ/mol) substantially exceeds those of the canonical Watson–Crick G:C (96.6 kJ/mol) and neutral C:C (68.0 kJ/mol) pairs,^8^ rationalizing the exceptional thermodynamic stability of the *i*-motif under acidic conditions. The central N3–H^+^–N3 hydrogen bond has been further characterized as exhibiting an asymmetric double-well potential, a property that distinguishes it mechanistically from conventional hydrogen bonds and reflects the shared but asymmetrically distributed proton between the two cytosine N3 atoms.^9^

Beyond the paramount contribution of pH-dependent C:C^+^ base pair formation, *i*-motif stability is modulated by a range of intrinsic sequence-dependent and extrinsic environmental factors across a broad span of pH conditions.^10^ These include interactions with ligands,^11^ sequence composition, C-tract length, and the number and nature of nucleobases within the *i*-motif loops,^6,12–14^ flanking nucleotides,^15^ as well as ionic strength and cationic effects.^16–18^ Conversely, molecular crowding, kinetic partitioning, and other environmental factors can favor *i*-motif formation even at neutral pH.^19–21^

From a biological standpoint, compelling evidence for the existence of *i*-motifs in the nuclei of living cells was obtained using an *i*-motif-specific antibody, iMab.^22^ C-rich sequences with high *i*-motif-forming propensity are enriched at centromeres, telomeres, and gene promoter regions, where they are proposed to function as recognition elements for transcription factors^23^ and participate in the control of gene expression and genomic stability.^23,24^

Elucidating the biological functions of *i*-motif structures requires a detailed understanding of the conformational dynamics of cytosine-rich single-stranded DNA. The three-dimensional architecture of non-canonical DNA structures is intimately coupled to their biological activity, making a quantitative description of their conformational transitions essential. Although the molecular mechanisms underlying non-B-DNA behavior have been extensively investigated, the folding and unfolding pathways of these structures frequently involve multiple intermediates and branched parallel routes rather than simple two-state transitions, and their full conformational characterization remains an open challenge. In this context, protein- and, in particular, α-HL nanopore electrophysiology has emerged as a powerful single-molecule platform for real-time, high-resolution single-molecule identification and DNA conformational dynamics, including non-canonical DNA structures.^25–33^

The α-HL nanopore platform has been successfully applied to probe diverse *i*-motif topologies. Burrows and co-workers employed this approach to investigate *i*-motif structures formed by the human telomeric repeat sequence,^34^ whereas Xi and co-workers combined single-molecule nanopore electrophysiology with molecular dynamics simulations to elucidate the structural determinants of *i*-motif stability.^35^ In a complementary approach, the sensing capability of the α-HL nanopore has been exploited to examine, at the single-molecule level, conformational changes in the human telomeric *i*-motif induced by interaction with anticancer compounds.^36,37^

In our laboratory, we previously employed a comparable α-HL nanopore platform to detect and characterize, at the single-molecule level, a model *i*-motif formed from a cytosine-rich single-stranded DNA sequence bearing a poly-2′-deoxyadenosine (dA_9_) tail at the 3′ terminus, with emphasis on its conformational dynamics, pH responsiveness, and interactions with short peptide nucleic acid (PNA) ligands.^38^

In the present work, we employed a DNA fragment derived from the promoter region of the *c*-Myc proto-oncogene, which encodes a multifunctional transcription factor that coordinates a broad spectrum of cellular processes in both normal physiology and disease.^39^ *c*-Myc transcription is largely governed by the P1 and P2 promoter sites,^40^ and upstream of the P1 regulatory region lies the NHE III_1_ element, which has been demonstrated to adopt both transcriptionally active and silenced conformations within the promoter.^41^ Importantly, the C-rich strand of the NHE III_1_ sequence (mycPy27) can fold into an *i*-motif secondary structure,^42^ whose formation *in vivo* appears to occur at near-neutral pH and may be induced by transcription-generated negative superhelical structures. Furthermore, the *i*-motif, known to form within the *c*-Myc promoter, has been shown to interact selectively with small peptide ligands that modulate *c*-Myc transcription in intact cells.^43^ Further insights into the regulatory architecture of the *c*-Myc promoter are needed to guide the rational design of novel ligands and potential therapeutic agents, including characterization of the *i*-motif-forming potential of the complementary strand.

To this end, we employed the 52-nucleotide cytosine-rich *c*-Myc promoter sequence (*c*-MycC52),^43^ which has been shown to be essential for efficient transcriptional activity from the P1 and P2 promoters.^44^ We resorted to α-HL nanopore-based single-molecule electrophysiology to investigate *c*-MycC52 *i*-motif conformational dynamics under the topological constraints imposed by the nanopore environment. The geometry of the α-HL pore—wherein the vestibule accommodates intact folded *i*-motif structures while the narrow constriction gates translocation until unfolding occurs—enables real-time resolution of conformational transitions between distinct topological states of the vestibule-captured DNA. Analysis of ionic current blockades produced by captured *c*-MycC52 reveals that, although the sequence adopts a fully folded *i*-motif at acidic pH, incremental pH variation from ∼6.53 to ∼4.76 drives rapid interconversion among distinct topological species. Continuous-time Markov chain modeling of the underlying current fluctuations provides a quantitative kinetic description of switching between these species, affording discrimination between closely related folding topologies. Complementarily, analysis of *i*-motif residence times within the α-HL vestibule at 1 and 3 M KCl yields detailed insight into the ionic strength dependence of *c*-MycC52 *i*-motif stability and dynamics under nanopore-imposed topological confinement.

## Experimental Section

The comprehensive list of reagents and details regarding sample preparation are presented in the Supporting Information material. The 52-base, single-stranded C-rich *c*-Myc promoter sequence (*c*-MycC52) (5′-CTT CTC CCC ACC TTC CCC ACC CTC CCC ACC CTC CCC ATA AGC GCC CCT CCC G-3′, molecular weight 15,374.9 g mol^–1^, purity ≥ 90%) was synthesized and purified by HLB Panagene Inc. (Republic of Korea). To investigate the ability of DNA fragments to fold into *i*-motifs in low pH electrolyte, UV–vis spectroscopy experiments were performed using a NanoDrop OneC spectrophotometer (Thermo Fisher Scientific, USA) (Supporting Information material). Nanopore electrophysiology measurements were undertaken as previously described,^38^ and are presented in detail in the Supporting Information material. Briefly, the lipid bilayer was formed across an aperture manufactured on a Teflon film, after pre-treating it with a mixture of hexadecane in *n*-pentane (1:10; v/v). Single α-HL nanopores were inserted into the stable lipid membrane by adding 0.5–8 μL of the wild-type α-HL monomeric stock solution to the grounded, *cis*-compartment, under continuous stirring, allowing heptameric pore assembly. For experiments involving asymmetric pH changes implemented during the same measurement series, the pH of the electrolyte in the *cis-* compartment, containing the DNA molecules, was adjusted to the desired value by the dropwise addition of predetermined volumes of HCl stock solution, as established by prior titration experiments. The *trans*-compartment electrolyte was maintained at near-neutral pH throughout the measurements. Unless otherwise stated, both compartments contained 3 M KCl and 10 mM HEPES. Alternatively, for measurements performed in either 1 or 3 M KCl, the electrolytes were buffered with 10 mM MES, with the pH adjusted at the start of the experiment to 5.33 in the *cis*-compartment and maintained at near-neutral values in the *trans*-compartment.

## Results and Discussion

### pH-Dependent *c*-MycC52 *i*-Motif Manifestation in High Salt Conditions at the Single-Molecule Level

In a first series of investigations, nanopore-based measurements were employed to probe the pH-induced *i*-motif folding of the *c*-MycC52 under the high-ionic-strength conditions used throughout this study, with structural transitions independently corroborated by UV–vis difference spectroscopy.^45^ (Figure S1). To this end, single-molecule ionic current recordings were performed to resolve the blockade signatures characteristic of pH- and voltage-dependent *c*-MycC52−α-HL interactions. We exploited the entropic trapping capability of the α-HL vestibule domain, which enables metastable capture of folded DNA structures and reports on their dynamic population in bulk solution.^25^

As shown in Figure 1Ia,b, at neutral pH (7.04), *cis*-added *c*-MycC52 is electrophoretically captured at the α-HL vestibule entrance, generating transient downward current spikes, attributable predominantly to translocation of unfolded ssDNA through the positively-biased nanopore. Rare events reflecting capture of folded *c*-MycC52 within the nanopore lumen were also detected (Figure 1Ic,IIb,c). Upon stepwise acidification via controlled HCl addition to reduce pH from 7.04 to ∼6.53 *within the same experiment*, intermediate-duration current blockades emerged more consistently at various transmembrane potentials applied from the amplifier (ΔVs) (substate *I*_V_, Figure 1IIa–f), consistent with the preferential interaction of the folded intramolecular *i*-motif structure^43^ with the α-HL vestibule. This result establishes that the high ionic strength of the electrolyte does not preclude *c*-MycC52 folding into an *i*-motif, consistent with prior observations.^46^

**Figure 1:**
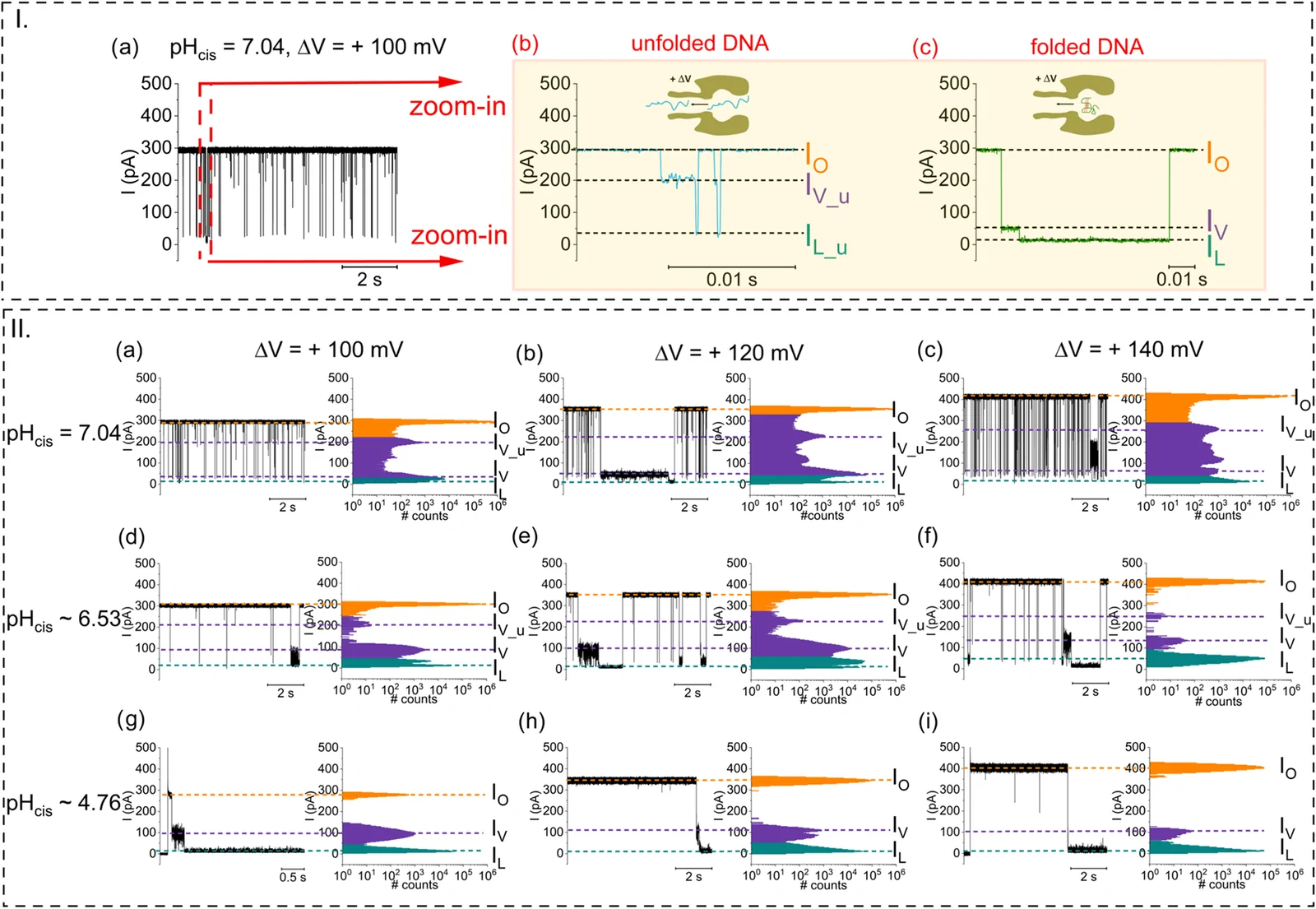
Single-molecule nanopore detection of pH-driven folding and unfolding of the *c*-MycC52 fragment. (I) (a) Representative trace displaying the capture of *cis*-added *c*-MycC52 [1 μM] at the vestibule entrance of a positively-biased α-HL (Δ*V* = +100 mV) in symmetrical 3 M KCl, 10 mM HEPES (pH = 7.04). The expanded segment in panel (b) shows zoomed-in views of the original recordings, highlighting the open-pore current (*I*_O_), transient dwelling of disordered (unfolded) *c*-MycC52 within the nanopore vestibule, which gives rise to the residual current *I*_V_u_, and subsequent translocation through the constriction into the lumen, manifested as a further reduction in ionic current to *I*_L_u_ (*I*_O_ = 294.1 ± 0.1 pA, *I*_V_u_ = 190.7 ± 6.0 pA and *I*_L_u_ = 31.7 ± 1.3 pA) (mean ± SEM). As revealed in panel (c), we occasionally observed similar blockade events, with the notable difference being a markedly reduced residual current during *c*-MycC52 occupancy of the α-HL vestibule (*I*_V_) and, subsequently, the lumen (*I*_L_), which we ascribe to capture of a folded *c*-MycC52 state, whereby *I*_V_ = 58.2 ± 16.0 pA and *I*_L_ = 20.9 ± 5.0 pA (mean ± SEM). The sketches in panels (b and c) illustrate the capture of the *cis*-added *c*-MycC52 fragment inside the nanopore, under the influence of the electrophoretic force indicated by the corresponding arrow. (II) Representative trace excerpts and corresponding all-point histograms acquired at the indicated transmembrane potentials, with *c*-MycC52 fragments [1 μM] added in the *cis*-side electrolyte at pH_cis_ = 7.04 (a–c), ∼6.53 (d–f), and ∼4.76 (g–i). The current-level notation is consistent with that used in panel (I) and indicates that, at mildly acidic pH (∼6.53), unfolded and partially folded *c*-MycC52 species coexist, as reflected by the concurrent observation of *I*_V_u_ and *I*_V_ blockade states.

The folding stability of *c*-MycC52 *i*-motifs was found to be acidity-dependent, as it entails a heterogeneous distribution of intermediate current levels and durations during capture into the nanopore. As revealed herein, at pH values up to ∼5.65 (see also Figure S2a–c), *I*_V_ intermediate blockade events proceeded via one of two distinct pathways: (i) a subsequent reversible deep current blockade (denoted *I*_L_—which we assign to a direct occlusion of the nanopore, as the vestibule-trapped fragment progressed to its constriction region leading to the nanopore lumen), followed by analyte release and return to the open-channel current level (*I*_O_), consistent with voltage-driven insertion of the *i*-motif into the constriction region, where it undergoes unfolding to a single-stranded conformation before traversing the nanopore (Figures 1IId–f and S2b); or (ii) direct return from the substate *I*_V_ to *I*_O_ without passage through *I*_L_, indicating that the folded *i*-motif was sufficiently stable within the vestibule to resist unfolding and instead exited through the *cis* entrance by which it had entered (Figures 1IId–f and S2c).

Noteworthy, experiments conducted at more acidic pH values (pH ∼4.76; Figure 1IIg–i, pH ∼4.43; Figure S2d–f, and pH ∼4.21; Figure S2g–i, respectively) revealed that events pertaining to substate *I*_V_ and consistent with *c*-MycC52 *i*-motif capture inside α-HL vestibule terminated in the irreversible deep current blockade level (*I*_L_). We ascribe this behavior to the formation of a known highly compact *c*-MycC52 intramolecular *i*-motif stabilized by the full complement of C:C^+^ base pairs,^43^ with additional stabilization potentially arising from electrostatic interactions with the positively charged inner surfaces of the vestibule and constriction regions,^47^ which collectively endow the structure with sufficient stability to remain lodged within the α-HL constriction and sustain long-lived pore occlusion. The apparent irreversible α-HL nanopore blockade by vestibule-captured *c*-MycC52 at low pH is consistent with prior findings establishing that, under acidic conditions, the melting temperature of *i*-motif structures increases with the number of C:C^+^ base pairs^48^—implying for our case that a higher degree of cytosine hemiprotonation lowers the folding free energy, stabilizes the folded state, and generates a more stable interaction between the α-HL constriction entrance and the DNA fragment.

### pH-Dependent Volumetric Analysis of *c*-MycC52 *i*-Motif Conformational States

We next employed volumetric analysis to probe the molecular-scale topology of the pH- and voltage-dependent conformational states of the *c*-MycC52 *i*-motif. We hypothesized that the increased stability of the *c*-MycC52 *i*-motif at lower pH values, evidenced by the persistence of the conductance substate corresponding to the vestibule-trapped *i*-motif (*I*_V_) (Figures 1IId–i, 2, and S2a–c), displays a pronounced dependence on both pH and Δ*V*. This behavior would likely stem from the competing destabilizing influences of electrophoretic and hydrodynamic forces exerted on the captured *i*-motif within the nanopore. This hypothesis is supported by prior single-molecule nanopore-based studies examining the pH dependence of *i*-motif lifetime under electrophoretic force, which established that *i*-motif residence time decreases with increasing pH, attributed to the higher probability of C:C^+^ base pair deprotonation at elevated pH values.^34^

**Figure 2:**
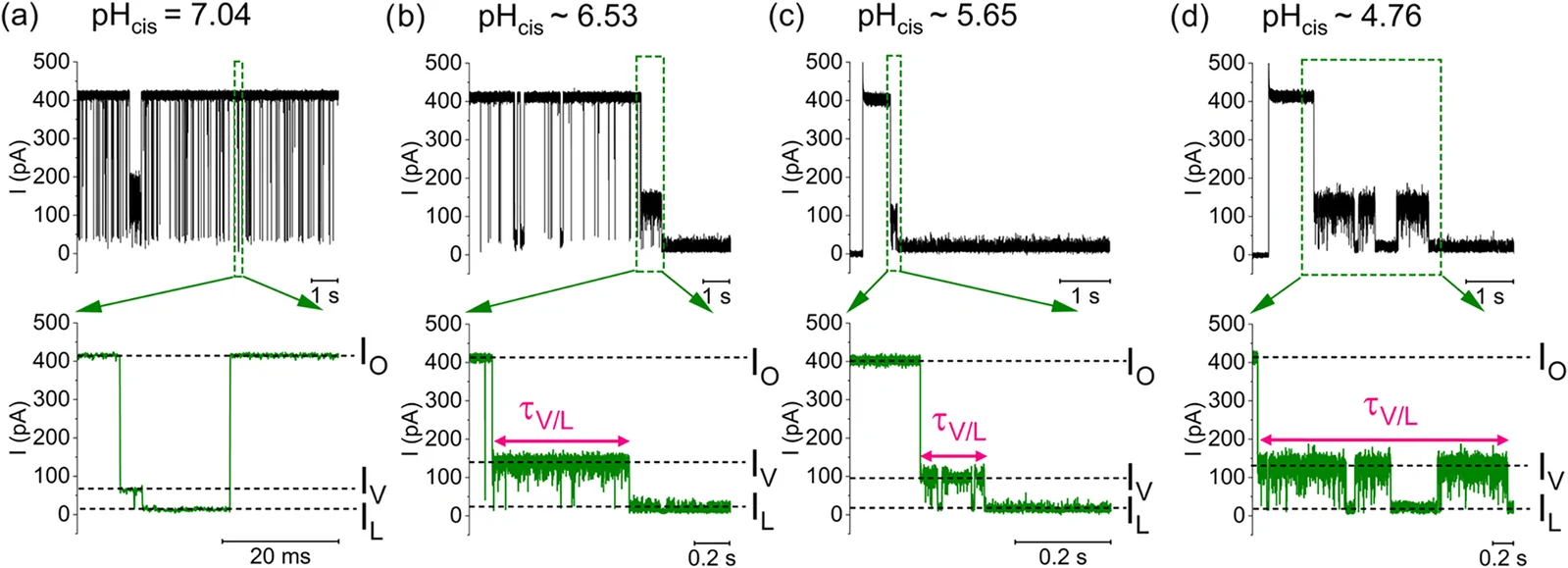
Entrapment of *c*-MycC52 within the α-HL vestibule generates a pH-dependent blockade fingerprint. Representative current traces and corresponding zoomed-in excerpts illustrating the capture of the *cis*-added *c*-MycC52 [1 μM] at the vestibule entrance of a positively-biased α-HL nanopore (Δ*V* = +140 mV). In these experiments, the pH of the *cis*-side electrolyte containing 3 M KCl and 10 mM HEPES set initially at 7.04 (a) (for brevity, only the rare folded *c*-MycC52 conformers are shown in this trace; the majority of blockades, resulting from unfolded *c*-MycC52, appear in Figure 1Ib), was varied stepwise within the same experimental run to attain the indicated values of pH_cis_ ∼6.53 (b), pH_cis_ ∼5.65 (c), and pH_cis_ ∼4.76 (d), under otherwise symmetric electrolyte conditions. The distinct conductive substates are denoted by *I*_O_ (open nanopore), *I*_V_ (*c*-MycC52 residing inside the vestibule), and *I*_L_ (*c*-MycC52 occluding the constriction region of the nanopore), respectively. In traces acquired under acidic conditions, τ_V/L_ is defined as the time between the onset of the vestibule blockade level (*I*_V_) and the near-total occluded state (*I*_L_).

In our analysis, the residual current measured upon *i*-motif fold capture inside the α-HL vestibule (*I*_V_) was normalized to the corresponding ionic current through the free, open nanopore (*I*_O_), to report on the relative blockade extent via $\frac{\Delta I_{V}}{I_{O}}=\frac{\left|I_{V}-I_{O}\right|}{I_{O}}$ (Figure 2), later used to estimate the molecular volume of the *i*-motif (δ).

To simplify quantitative volumetric estimations, the protein vestibule was viewed as a uniformly sized cylinder of length (*l*) and diameter (*d*), immersed in a buffer of electrical conductivity (σ), and clamped at a potential difference (Δ*V*). By disregarding the contribution of the access resistance to the nanopore, the volume (δ) of a captured analyte viewed as a cylinder aligned parallel to the electric field inside the nanopore of volume (*v*_vestibule_) that entails a relative current blockage of Δ*I*_V_ can be estimated as^25^

1 $$\delta =\frac{\Delta I_{V}{\left(l\right)}^{2}}{\sigma \Delta V}=\frac{\Delta I_{V}}{I_{O}}v_{vestibule}$$

Assuming an average value of *d* = 3.1 nm and *l* = 5 nm for the α-HL’s vestibule diameter and length, respectively, its volume was estimated at *v*_vestibule_ = 38 nm^3^.

As evidenced representatively in Figure 2, under acidic pH conditions, the folded *c*-MycC52 species became prevalent, and at pH ∼6.53, the estimated volume of the *c*-MycC52 *i*-motif was δ = 25.7 ± 0.5 nm^3^, while at pH ∼5.65 and ∼4.76, the vestibule-captured *i*-motif volumes converged to δ = 26.4 ± 0.2 nm^3^ and δ = 26.5 ± 0.5 nm^3^, respectively. The increased molecular volume in the acidic pH range, relative to linearized DNA at neutral pH (Figure 3f), is in line with the formation of a compact, globular *i*-motif stabilized by hemiprotonated C:C^+^ base pairs under acidic conditions. By contrast, at neutral pH, the less compact *c*-MycC52 topology is more susceptible to structural disorganization within the α-HL vestibule, manifesting as a correspondingly reduced molecular volume. In the calculations presented above, the molecular volumes resulted from a zero-slope linear fit of the corresponding δ vs Δ*V* plots, and this was justified by the apparent Δ*V*-independent relative nanopore blockades ( $\frac{\Delta I_{V}}{I_{O}}$ ) elicited by a vestibule-trapped *c*-MycC52 fragment (Figure 3a–e).

**Figure 3:**
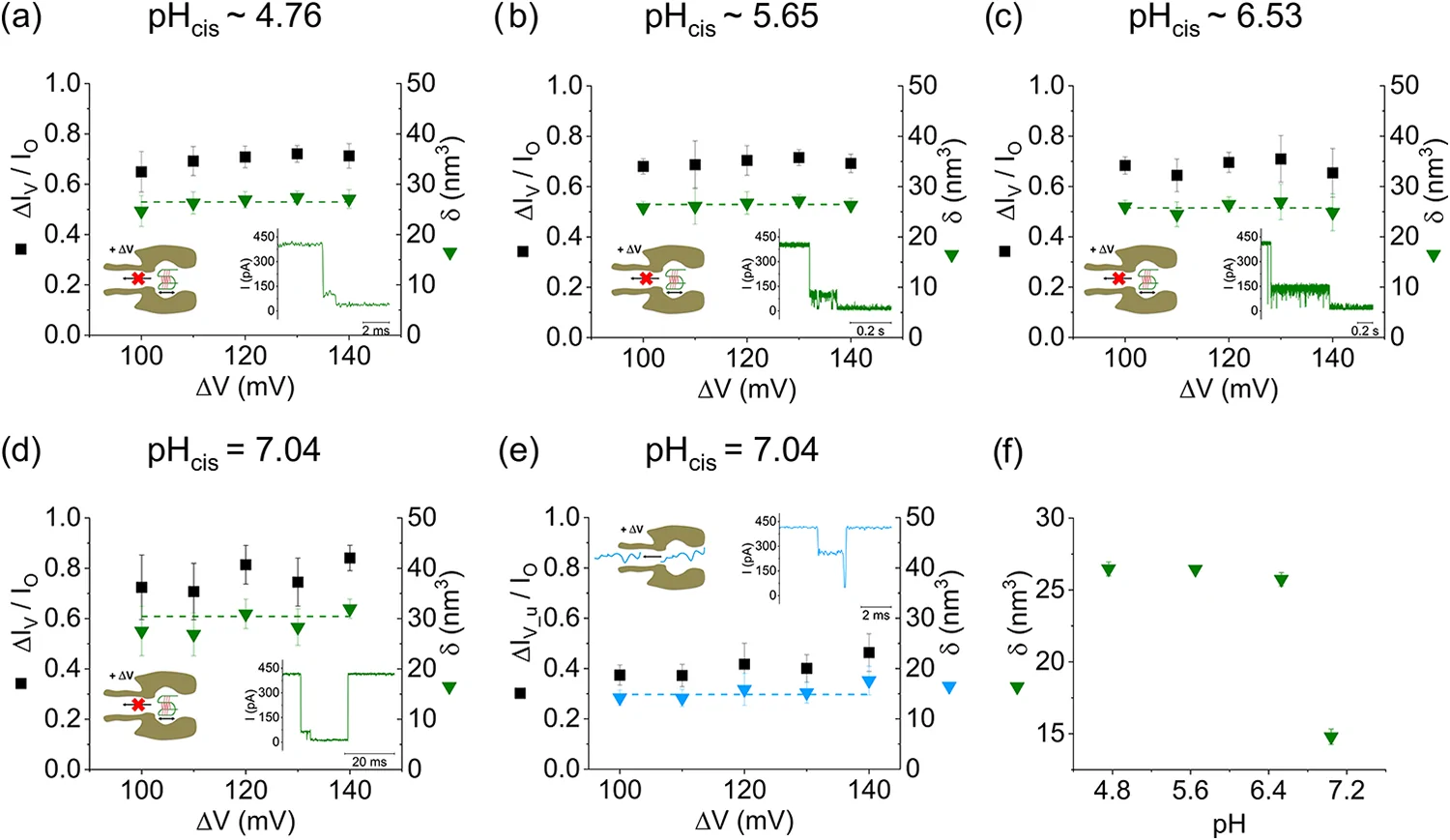
Structural compaction of *c*-MycC52 *i*-motif probed by nanopore volume measurements. Voltage dependence of the relative vestibule blockade generated at various pH_cis_ values by the folded species of *cis*-side added *c*-MycC52 [1 μM] (left axis: Δ*I*_V_/*I*_O_, black squares) (a–d), its unfolded conformer seen at neutral pH (e) (left axis: Δ*I*_V_u_/*I*_O_), and of the derived molecular volume δ (right axis, triangles) of folded species seen in acidic pH and respectively of the unfolded DNA around neutral pH. In each panel, the horizontal dashed line is the zero-slope linear fit (slope fixed to zero) of δ versus Δ*V*, whose constant value yields the molecular volume δ. Insets show representative current traces recorded at Δ*V* = +140 mV and the corresponding sketch of *c*-MycC52 capture inside the nanopore (green, folded *i*-motif; blue, unfolded *c*-MycC52), also suggesting that the folded *c*-MycC52 (panels a–d) does not translocate across the nanopore. (f) Molecular volume δ of the prevalent DNA species as a function of pH_cis_, reported as mean ± SEM.

Supplementarily, the analysis in Figure 3d,e reveals a pronounced difference between the estimated molecular volumes (mean ± SEM) of a folded *i*-motif-like species (presented initially in Figure 1Ic) (δ = 30.4 ± 0.9 nm^3^) and respectively linearized *c*-MycC52 traversing the α-HL vestibule at near-neutral pH (presented initially in Figure 1Ib) (δ = 14.01 ± 0.8 nm^3^), the former being only rarely observed under identical near-neutral conditions. As we detail in another chapter (vide infra), this folded species became totally disrupted at a slightly basic pH value.

As a cautionary note, it cannot be excluded that pH-determined volumetric changes of *c*-MycC52 in free solution may differ from the values estimated here, as confinement within the α-HL vestibule may impose an entropic barrier that modulates pH-driven folding transitions under applied voltage. The *c*-MycC52 sequence itself—comprising four cytosine-rich tracts of four cytosines each, interconnected by three five-nucleotide loops that organize the C:C^+^ base-paired core^43^—may further contribute to the topological constraints experienced within the nanopore. Nevertheless, the approach presented here offers an efficient and experimentally tractable strategy for real-time, single-molecule interrogation of the factors governing *c*-MycC52 *i*-motif folding and unfolding equilibria.

For simplicity, the analysis presented herein treats the *c*-MycC52−α-HL interaction as governed by a single composite physical scenario, in which dominant pH-dependent volumetric changes accompanying *i*-motif formation are lumped together with pH-dependent electrostatic interactions between the analyte and the nanopore. These electrostatic interactions arise from two sources: (i) protonation of adenine (position N1, p*K*_a_ ∼4.25) and cytosine bases (position N3, p*K*_a_ ∼4.6) at low pH, which reduces the net negative charge of the *c*-MycC52 ssDNA fragment, and (ii) pH-tunable charges on ionizable amino acid residues lining the nanopore inner surface.^47^ This approximation is adopted specifically for the kinetic analysis presented below, which does not disentangle the pH-dependent effects described above.

### Kinetic Analysis of the Conformational Stability of the *c*-MycC52 *i*-Motif within α-HL

We next investigated the kinetics of *c*-MycC52 *i*-motif capture within the α-HL vestibule as a function of pH and applied transmembrane voltage (ΔV), as a quantitative measure of conformational stability under varying solution conditions. As shown representatively in Figure 4, at pH ∼4.76, the vestibule-captured *c*-MycC52 *i*-motif generating the residual current *I*_V_, undergoes in certain instances unidirectional translocation under the applied electrophoretic force, leading to apparent direct occlusion of the nanopore constriction leading to the α-HL lumen. This results, after the time interval denoted by τ_V/L_, in a deep current blockade *I*_L_ (Figure 4Ia,b,IIa–e).

**Figure 4:**
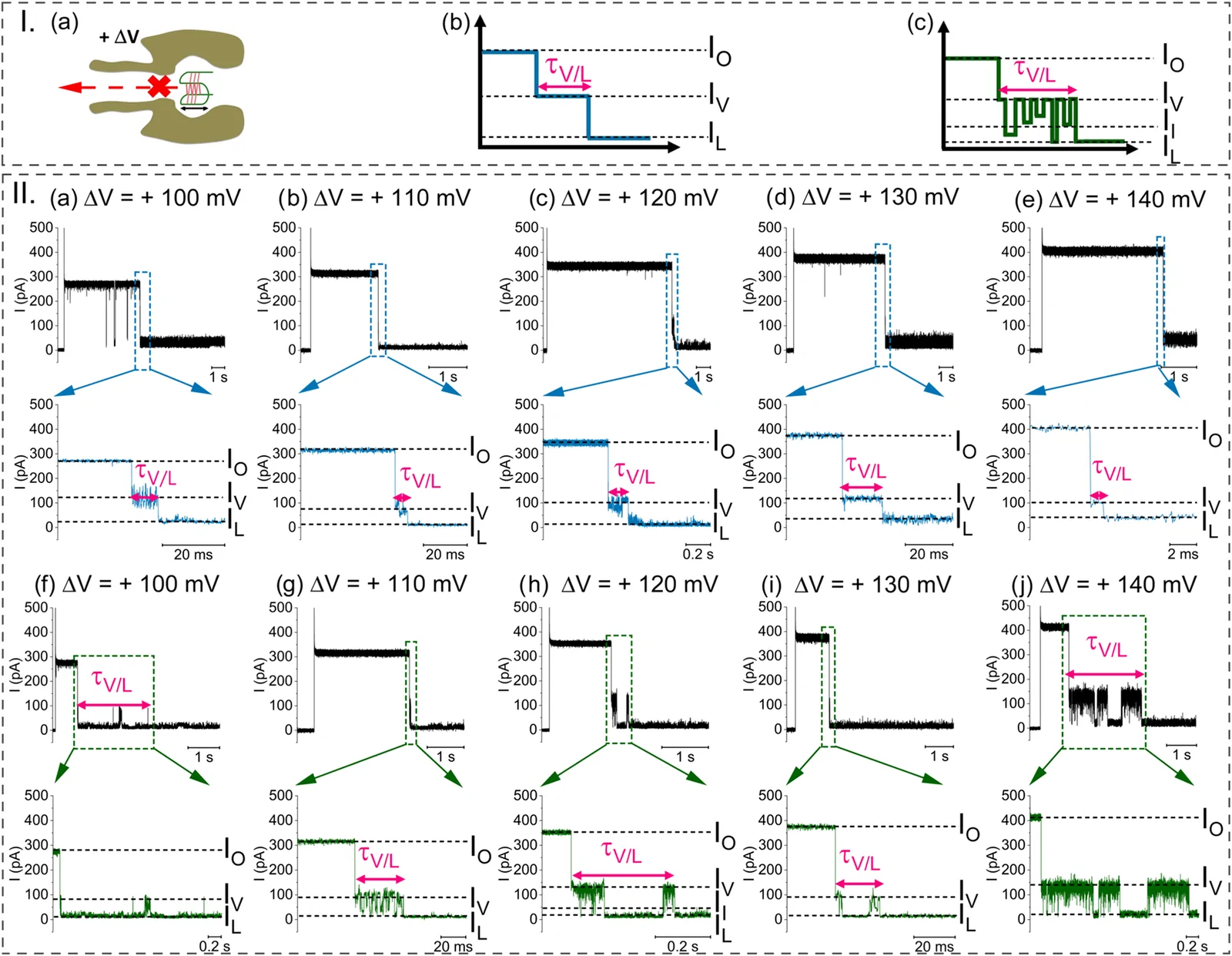
Direct and stepwise vestibule capture kinetics of the *c*-MycC52 *i*-motif in α-HL at acidic pH. (I) Schematic illustration of capture mechanisms; a folded *c*-MycC52 cannot translocate across the α-HL constriction (a) and is instead captured within the nanopore vestibule via either a direct pathway (b) or a stepwise pathway involving multiple intravestibular rearrangements (c). (II) Representative current traces of *cis*-added 1 μM *c*-MycC52 recorded in symmetrical 3 M KCl, 10 mM HEPES, with the *trans*-compartment held at pH = 7.04 and pH_cis_ ∼4.76, at applied potentials of +100 to +140 mV, showing direct (a–e) and stepwise (f–j) vestibule capture events. Insets show expanded views of the vestibule residence interval, τ_V/L_, used to quantify the kinetics of conversion from the vestibule-bound state (V) to the low-conductance state (L). Dashed lines indicate the open-pore current (I_O_) and the two blockade levels corresponding to vestibule entry (*I*_V_) and occlusion of constriction entrance to the lumen (*I*_L_).

Alternatively, the *c*-MycC52 *i*-motif generating the *I*_V_ current within the nanopore vestibule can reversibly access an intermediate substate (*I*), detected as *I*_I_ currents with broadly distributed amplitudes and dwell times, before proceeding to constriction occupancy and formation of the deeper blockade *I*_L_ (Figure 4Ic,IIf–j). In some instances, long-lived *I*_L_ events lead to irreversible pore obstruction, which was reversed by application of negative voltage to eject the *i*-motif into the *cis* chamber and restore full conductance.

Based on the additional data presented in Figures S3 and S4, which reveal a qualitatively similar kinetic behavior of the vestibule-captured *c*-MycC52 *i*-motif at acidic pH values, we propose that the intermediate substate *I*_I_ reflects subtle conformational interactions of the *c*-MycC52 with the inner surface of the α-HL vestibule. In this line of thought, once confined within the vestibule, the *i*-motif undergoes pH- and Δ*V*-dependent bending and stretching motions under the combined influence of electrophoretic and electroosmotic forces, together with electrostatic interactions with the vestibule inner surface, prior to occluding the constriction entrance to the β-barrel lumen—a state associated with near-zero residual current (*I*_L_).

Given that *i*-motif formation is favored over a narrow acidic pH range, the pH-dependent kinetic transitions from the initially captured *i*-motif in the α-HL vestibule (substate *I*_V_) to irreversible occlusion of the lumen entrance (substate *I*_L_), as evidenced by the data presented in Figures 4, S3, and S4, likely reflect the direct influence of H^+^ concentration in stabilizing the folded state through C:C^+^ base pair formation or, conversely, destabilizing it through disruption of cytosine hydrogen bonding. Importantly, therefore, the presented approach enables direct, real-time monitoring and quantification of *i*-motif conformational destabilization at the single-molecule level, a capability not readily accessible through conventional ensemble techniques.

As an initial assessment of the observed phenomenon, we quantified the experimental observable τ_V/L_, defined as the first-passage time required for the system to transition from a substate characterized by residual current *I*_V_ to substate *I*_L_ for the first time, following multiple reversible transits through substate *I*_I_ (Figures 4, S3, and S4). As shown in Figure S5, the V → L transition kinetics are fastest at pH ∼5.65 relative to both slightly lower (pH ∼4.76) and higher (pH ∼6.53) values, an effect most pronounced at applied transmembrane voltages of Δ*V* = +120, +130, and +140 mV. This indicates the presence of an optimal pH regime in which the *c*-MycC52 *i*-motif adopts a metastable conformational state that facilitates rapid voltage-driven progression from vestibule entry to constriction occlusion, whereas deviations toward either more acidic or near-neutral conditions reduce this kinetic efficiency, consistent with an altered balance between *i*-motif stability and electrophoretic/electroosmotic transport forces.

To obtain a more rigorous kinetic description of the vestibule-captured *c*-MycC52 *i*-motif, and on the basis of the experimental results presented above, we propose that the overall kinetic behavior of the captured *i*-motif is consistent with reversible transitions among three discrete substates—vestibule (V; 1), intermediate (I; 2), and lumen (L; 3)—governed by a continuous-time three-state Markov kinetic model represented as $\left(L\left(3\right)\overset{k_{VL}}{\leftarrow }V\left(1\right)\begin{array}{l} k_{VI}\\ ↔\\ k_{IV} \end{array}I\left(2\right)\right)$ . This simplified kinetic model is motivated by the critical experimental observations made at the single-molecule level, namely, upon capture of *c*-MycC52 *i*-motif within the α-HL vestibule generating substate (V) the fragment can either undergo a direct transition to the low-conductance substate (L) (Figures 4IIa–e, S3IIa–c, and S4Ia–e) or it accesses reversibly an intermediate substate (*I*), before progressing to constriction occlusion and formation of the lowest-conductance substate (L) state (Figures 4IIf–j, S3IIIa–e, and S4IIa–e).

To quantify the corresponding rate constants, we modeled the kinetic system within the framework of a homogeneous, continuous-time, discrete-state Markov chain with constant transition rates as indicated in the model above (see the Supporting Information, chapter “Current Fluctuation Analysis within the Continuous-Time Markov Chain Framework”). Given the limited number of dwell-time intervals obtainable within any single experiment—a consequence of the predominantly irreversible nanopore occlusion observed under low pH conditions—reliable statistical analysis required the assumption of ergodicity, or equivalently, time-translation invariance of the current signatures. Under this assumption, events pooled from multiple independent experiments were treated as randomly drawn excerpts from a single, statistically representative long-duration current trace of *i*-motif–nanopore interactions.

Based on experimentally accessible observables—specifically, the first-passage times for V → I transitions (τ_VI_) and intervals corresponding to V → L transitions (τ_VL_), I → V transitions (τ_IV_), and V → L transitions involving multiple intermediary visits (τ_V/L_) (Figure S6)—we calculated corresponding average time intervals (see the Supporting Information, Chapter “Current Fluctuation Analysis within the Continuous-Time Markov Chain Framework”), including: (i) the mean of τ_VI_ and τ_VL_ (denoted $\overset{®}{\tau_{VV}}$ ), (ii) the mean interval for first transitions from the intermediate substate (*I*) to the vestibule substate (V), quantified as $\overset{®}{\tau_{IV}}$ , and (iii) $\overset{®}{\tau_{V/L}}$ (Table S1). From these quantities, the transition rate constants *k*_VI_, *k*_VL_, and *k*_IV_ were uniquely determined (see the Supporting Information, Chapter “Current Fluctuation Analysis within the Continuous-Time Markov Chain Framework”) and reported in Table 1.

**Table 1:** pH-Dependent, Descriptive Kinetics of the Captured *c*-MycC52 *i*-Motif Inside α-HL at Δ*V* = +120 mV

	*k*_VI_ (s^–1^)	*k*_IV_ (s^–1^)	*k*_VL_ (s^–1^)
**pH**_**cis**_ **∼4.76**	243.1	409.8	3.3
**pH**_**cis**_ **∼5.65**	52.8	208.8	9.02
**pH**_**cis**_ **∼6.53**	41.8	28.7	2.9

To interpret these results in light of the limited structural information currently available on the *c*-MycC52 *i*-motif,^43^ we propose that the mean duration of the long-lived event ( $\overset{®}{\tau_{V/L}}$ ) as well as the *k*_VL_ reasonably reflects *i*-motif conformational stability during vestibule encapsulation, prior to the onset of a deep current blockade event (substate *I*_L_), in which the folded structure occludes the luminal β-barrel entrance without translocating. Based on the numerical evaluations undertaken at the selected pH_cis_ values of ∼6.53, ∼5.65, and ∼4.76 at a fixed transmembrane potential of Δ*V* = +120 mV (Table 1) or distinct Δ*V*s (Figure S5), we propose that around pH_cis_ ∼5.65, the fine interplay between the compacted topology of captured *i*-motif and charge–charge electrostatic interactions with the inner side of the vestibule and constriction, which become positively charged at acidic pHs,^47^ facilitates the electrophoretic force to determine the fastest pull of the *i*-motif structure against the constriction zone on the *trans* side of the vestibule.

Notably though, considering that the cytosine protonation state may vary significantly under our experimental conditions, e.g., at pH ∼4.76, approximately 50% of cytosine bases are expected to be protonated, whereas the protonated fraction should decrease at higher pH values, a relationship between cytosine protonation levels and the observed nanopore blockade signals might exist—as also underlined by single-molecule recordings presented herein (Figures 1, 2, and S2–S4), which might provide further insight into the mechanism of *i*-motif formation and stability, and we propose to investigate this in finer details in forthcoming work.

The pH-dependent *k*_VI_ and *k*_IV_ rate constant values, indicative of increasingly frequent interactions of the *i*-motif with the central constriction at lower pH, may reflect subtle differences in the size and shape of polymorphic *i*-motif folds arising from small energetic differences among distinct conformational intermediates that differ in their degree of C:C^+^ base pair formation,^49^ which modulate the molecular interactions at the interface between the *i*-motif and the α-HL interior surface.

In support of this interpretation, we recall that *i*-motif formation requires, among other structural prerequisites, protonation of cytosine N3 positions. Given that the p*K*_a_ of this group falls between 4.2 and 4.8—subject to variation with temperature and ionic strength—*i*-motif formation and stability are strongly pH-dependent, conferring considerable conformational flexibility on these structures across the pH range investigated here (pH ∼6.53, ∼5.65, and ∼4.76), with respect to both phosphate group orientation and the helical arrangement of successive cytidine residues. These findings extend the growing body of work on nanopore-based characterization of non-canonical DNA structures^34,50,51^ and demonstrate that α-HL-based electrophysiology constitutes a powerful single-molecule analytical platform for resolving the conformational topology distribution of *i*-motif structures, including highly dynamic and rapidly interconverting folding states under controlled physicochemical conditions, not readily achievable by conventional ensemble methods.

### Ionic Strength Dependence of *c*-MycC52 *i*-Motif Volumetric Properties and Conformational Stability

Although substantial literature supports the pivotal role of ionic conditions in modulating the *i*-motif structure and function, conflicting evidence still persists, with reports supporting either *i*-motif destabilization^48,52–56^ or stabilization^17^ with increasing ionic strength.

In other works, including efforts undertaken in our lab, reporting on single-molecule investigation of DNA fragments with the α-HL nanopore, it was firmly established that KCl concentrations as large as 1 M^34^ or 3 M^38,46^ do not preclude *i*-motif formation, underscoring that although cations compete with protons for the hemiprotonated C:C^+^ hydrogen bonds, pH remains the primary determinant of *i*-motif folding.^57^

Here, we extended this line of studies and aimed at gaining a more consistent picture about the effect of salt on the *c*-MycC52 *i*-motif. As shown in Figure S7, single-molecule experiments demonstrate that *c*-MycC52 *i*-motif structures form with comparable stability in the presence of either 1 or 3 M KCl at pH 5.33, as evidenced by the appearance of the irreversible deep current blockade event (substate *I*_L_) following reversible transitions between the vestibule-captured state (substate *I*_V_) and the intermediate substate (*I*_I_) within the vestibule. This result clearly excludes the possibility that at 3 M KCl, charge screening of the DNA backbone promotes unfolding into a flexible single-stranded conformation that, in turn, would preclude *c*-MycC52 *i*-motif formation.

Consistent with the observations at 3 M KCl (Figure 1I), nanopore experiments conducted at 1 M KCl and near-neutral pH (pH 7.03) revealed two distinct populations of blockade events: shorter events generating residual currents consistent with translocation of unfolded ssDNA across the α-HL, and longer events that, based on volumetric analysis, are consistent with the presence of a putative folded *i*-motif (Figure S8). At slightly basic pH (pH 8.13), *i*-motif-like blockade events were absent entirely, indicating complete disruption of the folded structure under these conditions. Together, these observations establish that *c*-MycC52 retains the capacity to fold into an *i*-motif-compatible structure at near-neutral pH, albeit with substantially lower propensity than under acidic conditions (Figure S8).

The influence of K^+^ concentration on *c*-MycC52 conformation was assessed by exposing the DNA to an electrolyte containing either 1 or 3 M KCl at pH 5.33, a condition previously established to promote *i*-motif folding. Residual currents arising from *i*-motif capture within the nanopore vestibule (substate *I*_V_) were normalized to the open-nanopore current (*I*_O_) to quantify the relative blockade extent during vestibule occupancy, and subsequently used to estimate the molecular volume (δ) of the captured *i*-motif. The resulting molecular volumes at the two ionic strengths were comparable, with a marginally more compact structure observed at 1 M KCl (δ = 26.4 ± 0.32 nm^3^) relative to 3 M KCl (δ = 27.2 ± 0.5 nm^3^) (Figure S9).

From a kinetic standpoint, we quantified here the first-passage time intervals τ_V/L_, which, as defined above, measure the time required for the system to transition from substate *I*_V_ to substate *I*_L_ for the first time, following multiple reversible transits through substate *I*_I_ (Figure S7, c, d). Statistical analysis of these intervals revealed faster progression to the deep blockade state (substate *I*_L_) at 1 M KCl (τ̅_V/L_ = 0.18 ∓ 0.05 s) (mean ± S.D.) than at 3 M KCl (τ̅_V/L_ = 1.2 ∓ 0.3 s) (mean ± S.D.), indicating that the vestibule-captured *i*-motif engages the constriction more rapidly at lower ionic strength. This finding indicates that although molar K^+^ concentrations (1 and 3 M KCl) do not preclude *c*-MycC52 folding into *i*-motif structures of largely similar molecular volumes at pH 5.33, a reduced ionic strength (1 M vs 3 M KCl) favors a conformationally restricted folded structure that more effectively engages and occludes the α-HL constriction, yielding substate *I*_L_. This conformationally restricted folded structure of the *c*-MycC52 *i*-motif, favored at 1 M relative to 3 M KCl, may be interpreted as reflecting a destabilizing effect of elevated ionic strength on intramolecular C:C^+^ base pairing, most likely mediated through the influence of cations on both the p*K*a of cytosine N3 and the salt-dependent electrostatic environment of the negatively charged phosphate backbone.^52^

## Conclusions

In summary, we demonstrate that the α-HL nanopore possesses sufficient sensitivity to discriminate and characterize the highly dynamic conformational equilibria of *c*-MycC52 *i*-motif structures under acidic conditions and across distinct voltages and ionic strengths. By monitoring *c*-MycC52 *i*-motif conformational dynamics in real time through single-molecule electrophysiology and continuous-time Markov chain kinetic modeling, we reveal that under the low pH conditions favoring cytosine hemiprotonation, polymorphic *i*-motif folds exhibit highly dynamic interconversion, driven by small energetic differences among conformational intermediates that differ in their degree of C:C^+^ base pair formation—differences that modulate molecular interactions at the interface between the *i*-motif and the α-HL interior surface and generate distinct ionic current fluctuation signatures. Furthermore, although *c*-MycC52 folds into *i*-motif structures of comparable molecular volume at both 1 and 3 M KCl under acidic conditions, the vestibule-captured *i*-motif exhibits greater kinetic stability at lower ionic strength. Overall, these results establish the α-HL nanopore as a versatile single-molecule platform for real-time characterization of the conformational dynamics of *c*-Myc promoter *i*-motif structures under topological confinement. This approach holds significant potential for probing *i*-motif stability across physicochemical conditions relevant to the intracellular environment, as well as for interrogating ligand recognition interactions—capabilities that may contribute to the rational design and rigorous evaluation of *i*-motif-targeted therapeutic agents.

## Supplementary Material



## Notes

Additional experimental details, materials, and methods, including tables and supplementary figures (PDF)
