# Supplementary material for: Probing the pH- and Voltage-Driven Intramolecular Folding Dynamics of the c‑MycC52 Promoter i‑Motif at the Single-Molecule Level
Source: Anal Chem. 2026 Sep 17;98(38):28475–84. doi: 10.1021/acs.analchem.6c04361 (PMC13629166; doi:10.1021/acs.analchem.6c04361)
Supplement: Supplementary file 1 [file ac6c04361_si_001.pdf]

Supporting Information for:

Probing the pH- and Voltage-Driven Intramolecular Folding Dynamics of the c-MycC52 Promoter i-Motif at the Single-Molecule Level

Adina Cimpanu[1,*], Jonggwan Park[2,*], Yoonkyung Park[2,#], Loredana Mereuta[1,#] Tudor Luchian[1,#]

[1]Department of Physics, Alexandru I. Cuza University, 700506 Iasi, Romania

[2]Department of Biomedical Science and Institute for Peptide Drugs (IPD), Chosun University, Gwangju 61452, Republic of Korea

[#]Corresponding authors: luchian@uaic.ro, loredana.mereuta@uaic.ro, y_k_park@chosun.ac.kr

*These authors contributed equally

Table of Contents

Reagents S-3

Sample preparation S-3

Spectral analysis of pH-dependent i-motif formation S-3

Nanopore electrophysiology S-4

Supplementary Figure S1 S-6

Supplementary Figure S2 S-7

Supplementary Figure S3 S-8

Supplementary Figure S4 S-9

Supplementary Figure S5 S-10

Current fluctuations analysis within the continuous-time evolution of a Markov chain framework S-11

Supplementary Figure S6 S-11

Table S1 S-18

Supplementary Figure S7 S-19

Supplementary Figure S8 S-20

Supplementary Figure S9 S-21

References S-22

Reagents. Potassium chloride (KCl), ultrapure water (DNase- and RNase-free), HEPES (N-(2-hydroxyethyl)piperazine-N′-(2-ethanesulfonic acid)), Tris (hydroxymethylaminomethane), and MES (2-(N-morpholino)ethanesulfonic acid) buffers, as well as HPLC-grade n-pentane, hexadecane, and wild-type α-hemolysin (α-HL) monomeric protein, were obtained from Sigma-Aldrich (Germany). 1,2-diphytanoyl-sn-glycerophosphocholine (DPhPC) lipids were purchased from Avanti Polar Lipids (Alabaster, AL, USA).

Sample preparation. Lyophilized DNA samples were dissolved in TE buffer (10 mM Tris, 1 mM EDTA, pH 7.9) containing 1 M NaCl to yield a 100 μM stock solution. The solution was vigorously stirred at 1,400 rpm for 3 min using a vortex mixer (Stuart BioCote, Sigma-Aldrich, Germany), heated to 95 °C in a digital block heater (IKA, Cole-Parmer, USA), and then slowly cooled to 22 °C to ensure proper rehydration and annealing. Aliquots for electrophysiology experiments were transferred to fresh vials, while the remaining stock solutions were stored at −20 °C until further use. Prior to each experiment, aliquots were subjected to the same heating–cooling cycle (95 °C to 22 °C) to maintain consistent conformational states.

Spectral analysis of pH-dependent i-motif formation. In such assays, 1 mL of a reference solution containing 3 M KCl buffered with 10 mM HEPES around pH = 7 was pipetted in a quartz cuvette with a 10-mm path length, and the absorption spectra were recorded in the 190–850 nm wavelength range at a room temperature of ~ 22° C. Subsequently, an appropriate volume from a DNA stock solution (200 μM) were added, to attain a final concentration of 0.5 μM DNA in the cuvette. Absorption spectra were recorded in the similar wavelength range around neutral pH and upon lowering incrementally the pH in the buffer to different values, by adding pre-determined volumes from a HCl stock solution, checked via pH titration measurements for the ability to set the acidity as desired. Then, we simply took the arithmetic difference between the UV-vis

spectrum in the unfolded state around neutral pH and the folded state at the various pH values set in the quartz cuvette with DNA present, to construct UV-vis difference spectra. To assess i-motif formation, we sought the characteristic emergence of a minimum at ~295 nm and a maximum at ~240 nm.

Nanopore electrophysiology. Bilayer chambers were separated by a 25-µm-thick Teflon film (Goodfellow, Malvern, MA, USA) containing an aperture of approximately 120 µm in diameter. In several experiments, manufactured Teflon septa with an 80-µm aperture and a dedicated Montal–Müller bilayer chamber system (ELEMENTS SRL, Italy) were used. All recordings were performed on a vibration-isolated platform (BenchMate 2210, Warner Instruments, USA) housed in a Faraday cage (Warner Instruments, USA) at room temperature (~22 °C). Ionic currents were measured in voltage-clamp mode using an Axopatch 200B or Multiclamp 700B amplifier (Molecular Devices, USA). Signals were digitized at a sampling rate of 50 kHz using a NI PCI 6221 16-bit acquisition board (National Instruments, USA) and low-pass filtered at 10 kHz or 12 kHz. In some cases, an ePatch amplifier (ELEMENTS SRL, Italy) was used for data acquisition and analysis. Current blockade events were analyzed using statistics of exponentially distributed events in pClamp 6.03 (Axon Instruments, USA). All reported data and statistics are derived from 3–6 independent experiments.

A proton gradient across the nanopore generates a diffusion potential, that alters the net transmembrane potential difference (ΔV) sensed by the nanopore. In the absence of a supporting electrolyte – for instance, if media 1 and 2 differed only in HCl concentration - such pH and correspondingly proton concentration (C) gradients would generate a sizable diffusion potential across the nanopore ($\Delta V_{diff.}$), with magnitude depending on the temperature (T), zF product where F is the Faraday constant and z is the modulus of monovalent ions, dissociated acid ion electric

mobilities (u_{Cl^-}, u_{H^+}), the gradient between the two media ($\frac{C_2}{C_1}$) and universal gas constant (R), according to $\Delta V_{diff.} = \frac{u_{Cl^-} - u_{H^+} RT}{u_{Cl^-} + u_{H^+} zF} ln \frac{C_2}{C_1}$.[1] Under the present experimental conditions, the concomitant presence in both chambers of dissociated salt ions at a far exceeding concentration than that of protons, and with nearly equal electric mobilities (the electrical mobilities of potassium K^+ and chloride Cl^- ions in dilute aqueous solutions at 25°C are nearly equal, with K^+ at approximately 7.6×10^{-8} $m^2V^{-1}s^{-1}$ and Cl^- at 7.7×10^{-8} $m^2V^{-1}s^{-1}$), results in their electrodiffusion across the interface where the proton-gradient-induced diffusion potential forms. In turn, dissociated salt ions electrodiffusion dominates the total ionic current across the nanopore, their presence suppresses the local charge asymmetry generated by the pH gradient, thereby offsetting the pH-gradient-derived diffusion potential (data not shown).

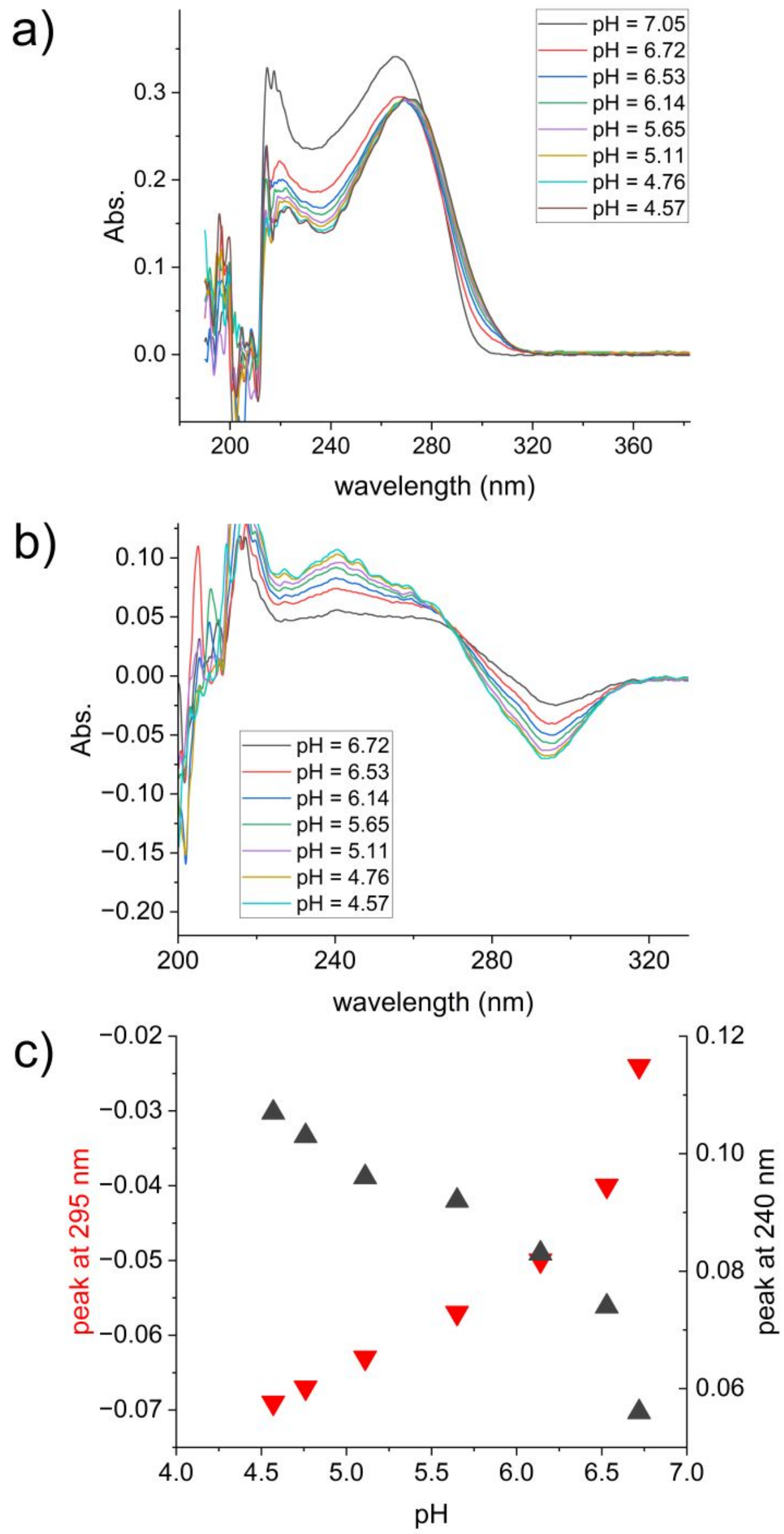

Figure S1. **Cytosine-rich c-MycC52 fragments with i-motif-forming propensity exhibit distinct UV absorbance profiles in their folded and unfolded states.** The thermal difference between the UV-vis spectrum in the unfolded state at pH = 7.05 (a) and the folded state at various acid pH values, as indicated for the c-MycC52 [0.5 μM] bathed in 3 M KCl and 10 mM HEPES (b), showing a minimum at ∼295 nm and a maximum at ∼240 nm (c), is a characteristic of i-motif folds depending on the pH.

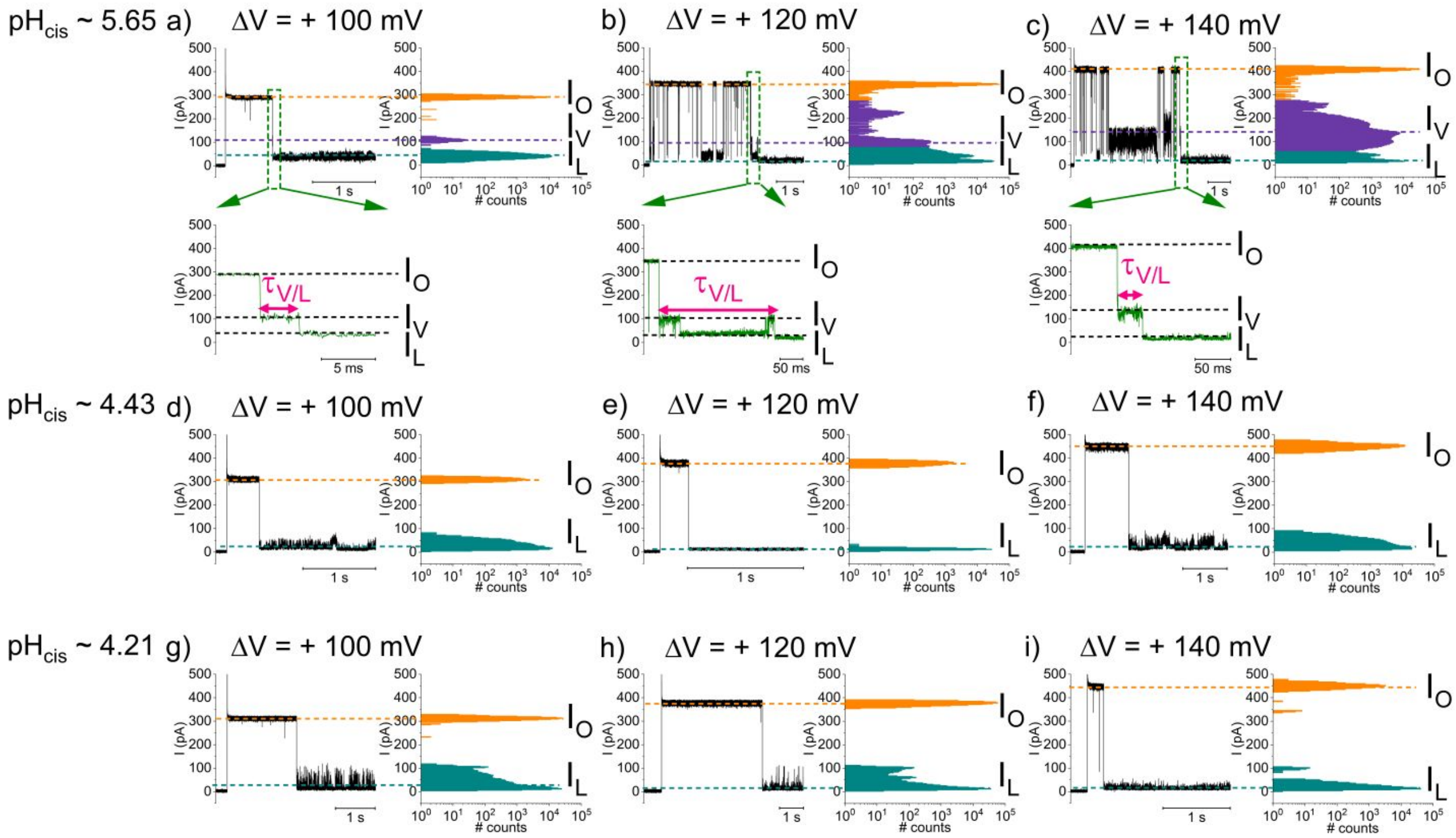

Figure S2. **Lower pH values promote the irreversible α-HL blockade by vestibule-captured c-MycC52**. Representative trace excerpts and corresponding all-point histograms acquired at the indicated transmembrane potentials, with c-MycC52 fragments [1 μM] added in the cis-side electrolyte at pH_{cis} ~ 5.65 (a–c), ~ 4.43 (d–f) and ~ 4.21 (g–i) measured in 3 M KCl, and 10 mM HEPES. To enable pH-dependent measurements within the same experiment, the cis electrolyte containing c-MycC52 (initially pH = 7.04) was acidified stepwise via successive additions of pre-determined volumes of HCl. As presented in the zoomed-in excerpts (green) from selected traces at pH_{cis} ~5.65 (panels a–c), both blockade levels (I_V and I_L) are clearly resolved, indicating a unidirectional $I_O \rightarrow I_V \rightarrow I_L$ transition. For brevity, $\tau_{V/L}$ denotes the dwell time between vestibule entry (I_V) and occlusion of constriction entrance to the lumen (I_L). Notably, at pH_{cis} ~4.43 (panels

d–f) and ~4.21 (panels g–i), the I_V vestibule level current blockade is no longer distinguishable from I_L, collapsing into the noise floor, consistent with rapid occlusion of the nanopore constriction by the captured c-MycC52 i-motif fragment.

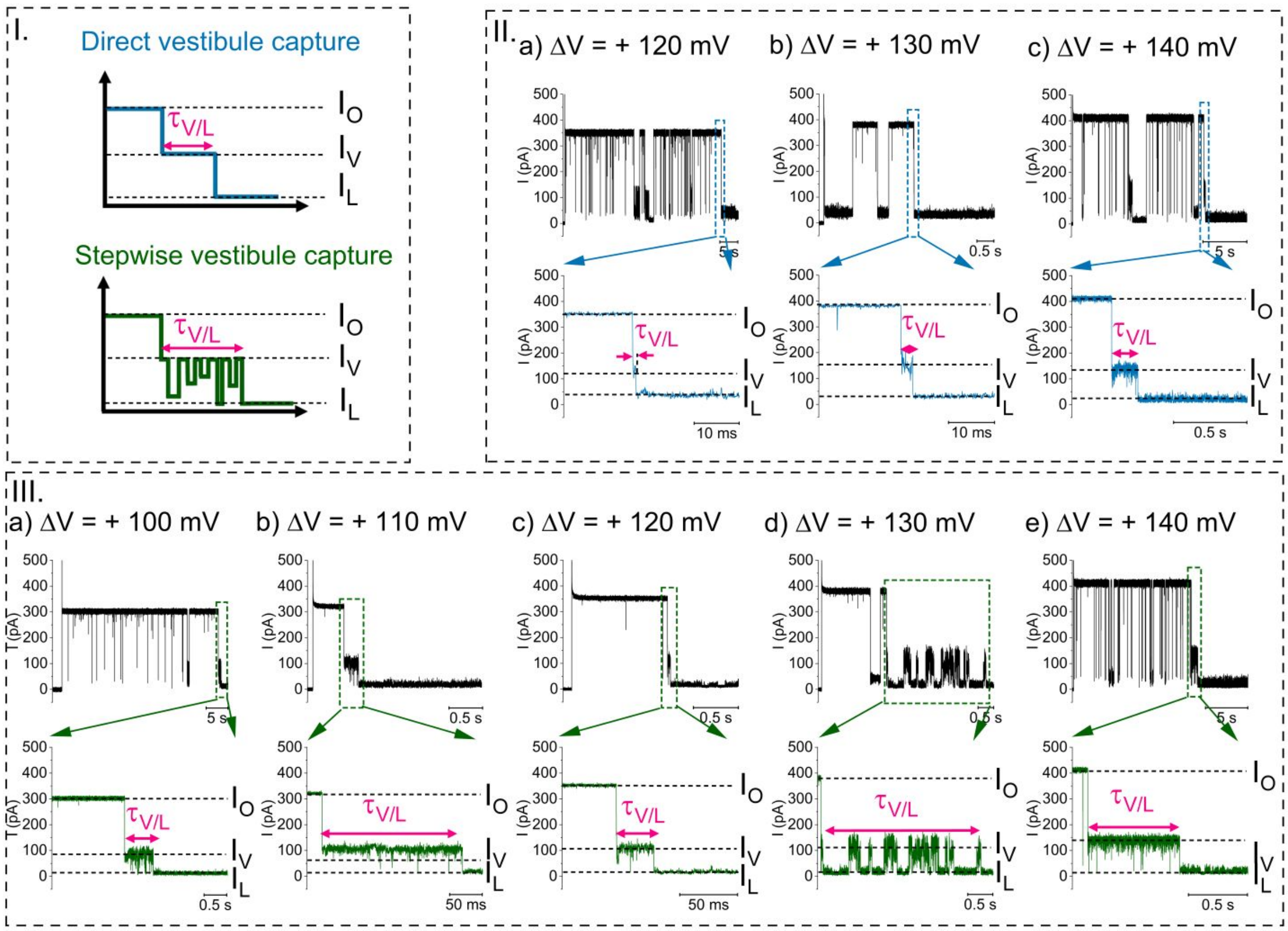

Figure S3**. Two distinct vestibule-capture signatures of the c-MycC52 i-motif at pH_{cis} ~ 6.53.** (I) Schematic representation of current traces illustrating direct vestibule capture and stepwise vestibule capture scenarios, both resolving I_O, I_V and I_L levels; $\tau_{V/L}$ denotes the time interval between vestibule entry (I_V) and occlusion of constriction entrance to the lumen (I_L). (II) Direct

vestibule capture events at ΔV = +120 mV (a), +130 mV (b), and +140 mV (c); representative single-channel traces and zoomed-in excerpts of cis-added c-Myc52 DNA [1 μM] captured by a positively biased α-HL nanopore in symmetrical 3 M KCl, 10 mM HEPES, with the trans compartment maintained at pH 7.04. The $pH_{cis} \sim 6.53$ was achieved by stepwise HCl addition. (III) Stepwise vestibule capture events at ΔV = +100 mV (a), +110 mV (b), +120 mV (c), +130 mV (d), and +140 mV (e). In all excerpts, dashed lines indicate I_O, I_V and I_L current levels.

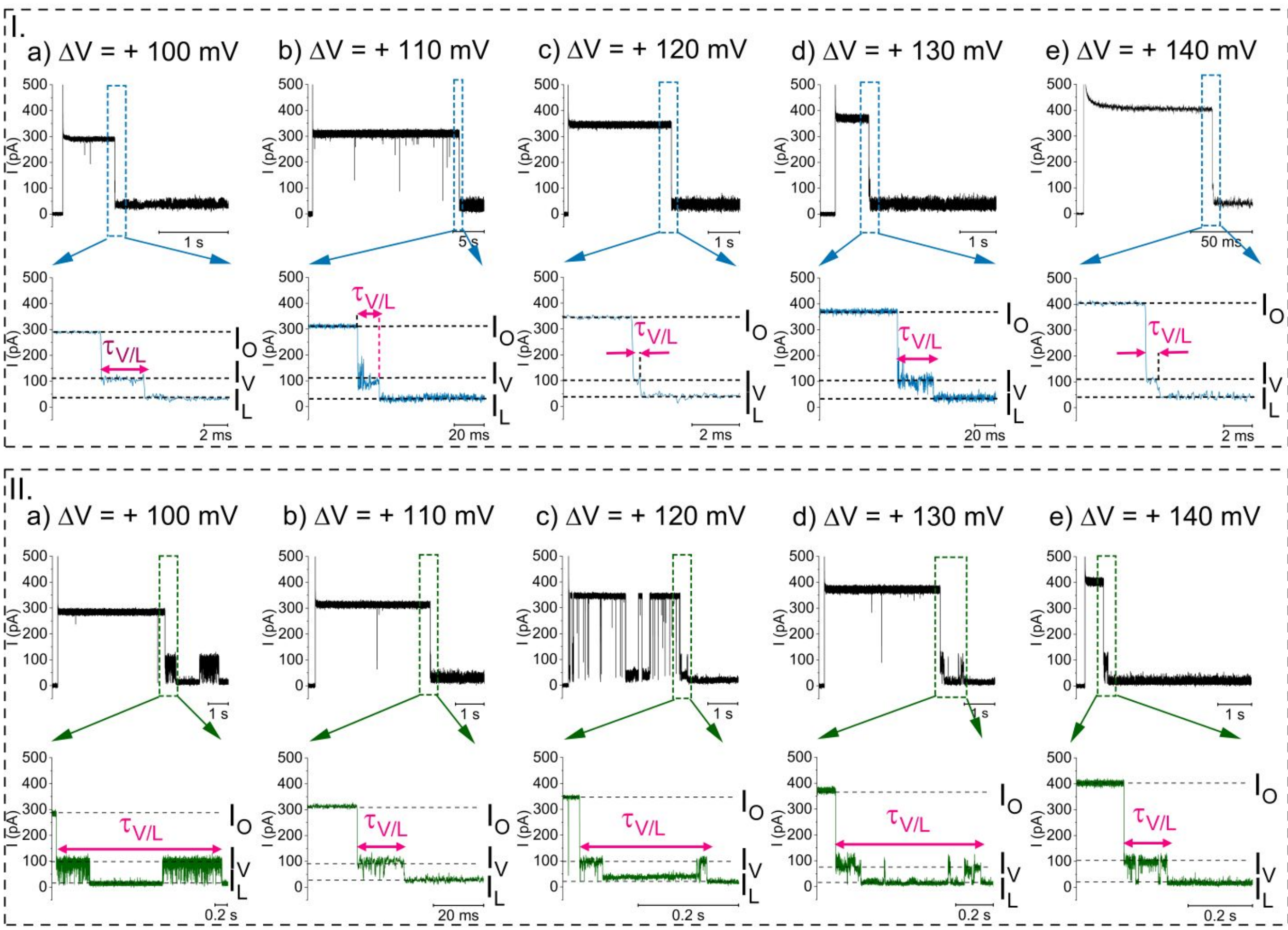

Figure S4. **Two distinct vestibule-capture signatures of the c-MycC52 i-motif at $pH_{cis} \sim 5.65$.** Representative single-channel traces and zoomed-in excerpts of cis-added 1 μM c-Myc52 DNA

captured by a positively biased α-HL nanopore in symmetrical 3 M KCl, 10 mM HEPES, with the trans compartment maintained at pH 7.04. The pH_{cis} ~ 5.65 was adjusted by stepwise HCl addition during the same experiment. Section I illustrates direct vestibule capture events at ΔV = +100 mV (a), +110 mV (b), +120 mV (c), +130 mV (d), and +140 mV (e), with excerpts highlighted in blue. Section II shows stepwise vestibule capture events at the same applied potentials (a–e), with excerpts highlighted in green. In all panels, dashed lines indicate I_O, I_V and I_L current levels; $\tau_{V/L}$ denotes the time interval between vestibule entry (I_V) and occlusion of constriction entrance to the lumen (I_L), and is indicated for each event.

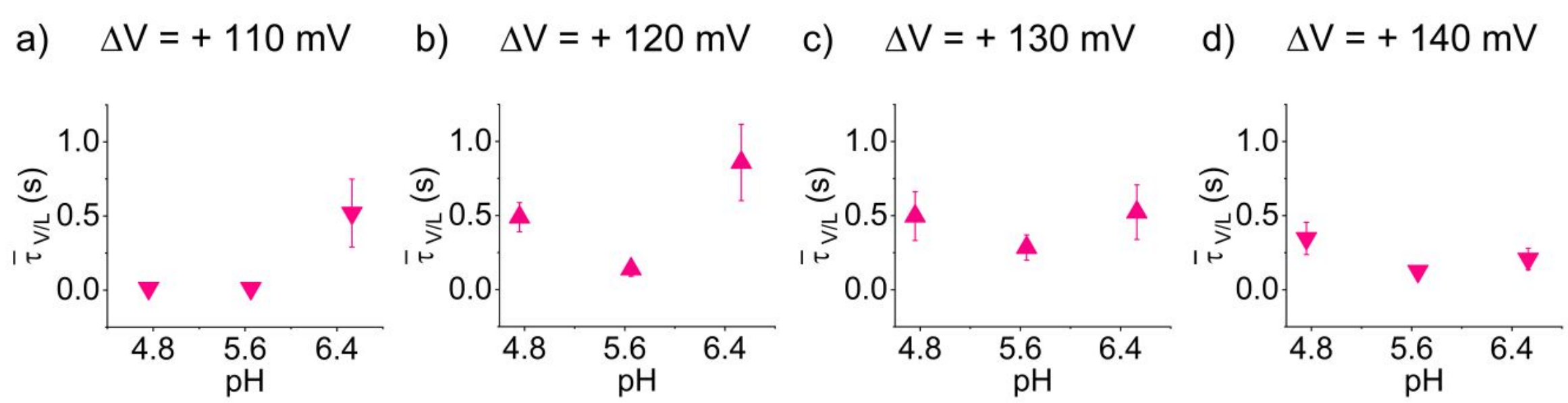

Figure S5**. Dependence of vestibule-capture kinetics on pH and applied voltage.** Average vestibule lifetimes, $\grave{\tau}_{V/L}$, of c-MycC52 i-motif trapped in the α-HL vestibule, measured at pH_{cis} ~ 4.76, ~ 5.65, and ~ 6.53 under applied potentials of (a) ΔV = +110 mV, (b) +120 mV, (c) +130 mV, and (d) +140 mV. The non-monotonic pH dependence reflects changes in the conformational stability and vestibular dynamics of the i-motif structure.

Current fluctuations analysis within the continuous-time evolution of a Markov chain framework

Regarding the kinetic analysis of the entrapped c-MycC52 i-motif in the α-HL vestibule nanocavity, experimental data provided an important clue on the putative, underlying kinetic model. As displayed representatively in Figure S6, the vestibule-captured c-MycC52 i-motif fragment generating the I_V electric current through the nanopore visits reversibly an intermediary substate (denoted by 'I') probed experimentally by the corresponding electric currents I_I before occupying the constriction entrance leading to the β-barrel, generating to a long dwell time and a deeper current blockage denoted by I_L.

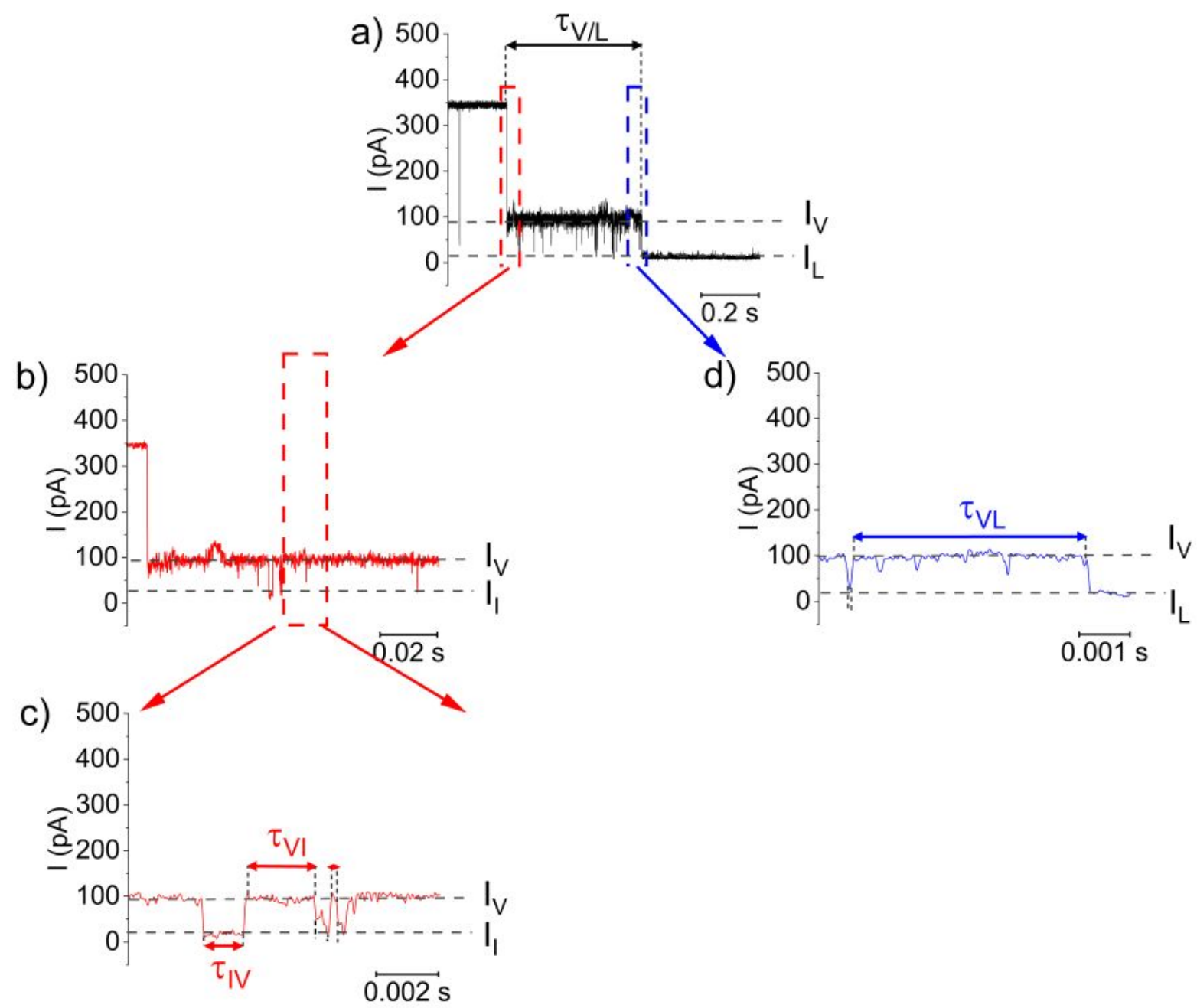

Figure S6. **Illustrative depiction of the kinetic unfolding pathway of a c-MycC52 i-motif entrapped within the α-HL vestibule.** Representative electrophysiological trace recorded at ΔV = +120 mV in 3 M KCl, 10 mM HEPES, with pH_{trans} = 7.04 and pH_{cis} ~ 6.53 adjusted by addition

of a predetermined volume of HCl. The recording shows: (a) an irreversible O → V → L transition of a captured c-MycC52 i-motif; (b, c) reversible transitions between the vestibule-bound (V) and intermediate (I) substates (V ↔ I); and (d) a direct V → L transition. The experimentally accessible dwell times and first-passage intervals associated with these stochastic transitions are indicated.

The long-lived blockade events (I_L) led in certain cases an irreversible obstruction of the nanopore, and a negative voltage was applied to return the i-motif to the cis chamber rendering the nanopore fully conductive. Although speculative at the moment, it cannot be excluded that the observed distinct blockade events induced by the vestibule-lodged c-MycC52 i-motif may originate from conformational transitions of the fragment itself while interacting with the interior surface of the α-HL nanopore. To further investigate this, we aimed at quantifying the observed transitions, by modeling the kinetic system within the framework of homogeneous, continuous-time, discrete Markov chain with constant reaction rates. As argued above, the experimental recordings provide strong indications that a three-states kinetic model describing a vestibule-lodged c-MycC52 i-motif-nanopore interactions may be depicted as in (1):

$$L(3) \xleftarrow{k_{VL}} V(1) \underset{k_{IV}}{\overset{k_{VI}}{\leftrightarrow}} I(2) \qquad (1)$$

As established, the time-dependent behavior of conditional transition probabilities between substates is described by the Chapman–Kolmogorov equations.[2] Applied for our case (Scheme 1, with the corresponding substates enumerated 1-3, as shown), the continuous-time evolution of a Markov chain with three substates for which transitions between any pair of distinct states are described by the shown corresponding transition rates, and can be quantified via the conditional probability matrix (P) which writes as:

$$P(t) = \begin{pmatrix} p_{VV}(t) & p_{VI}(t) & p_{VL}(t) \\ p_{IV}(t) & p_{II}(t) & p_{IL}(t) \\ p_{LV}(t) & p_{LI}(t) & p_{LL}(t) \end{pmatrix} \qquad (2)$$

where each $p_{ij}(t)$ term represents the conditional probability to be in state j at time t, starting from i ($p_{ij}(t) = Probability$(state j at time t|started in state i at $t = 0$).

In a compact form, the time-evolution of the system characterized by the Scheme 1 is the solution of the differential matrix equation:

$$\frac{d}{dt}P(t) = P(t)\,Q \qquad (3)$$

with initial condition:

$$P(0) = I_n(\text{n} \times \text{n identity matrix}) = P(t) = \begin{pmatrix} 1 & 0 & 0 \\ 0 & 1 & 0 \\ 0 & 0 & 1 \end{pmatrix} \qquad (4)$$

where the rate matrix (Q) writes:

$$\text{Q} = \begin{pmatrix} -k_{VI} - k_{VL} & k_{VI} & k_{VL} \\ k_{IV} & -k_{IV} & 0 \\ 0 & 0 & 0 \end{pmatrix} \qquad (5)$$

In (5), k_{VI}, k_{IV}, and k_{VL} denoting the reaction constants describing vestibule (V) → intermediary substate (I), intermediary substate (I) → vestibule (V) and respectively vestibule (V) → lumen (L) i-motif transitions. By solving Equation (3), one obtains the conditional probabilities $p_{ij}(t)$ of transitioning from substate i to substate j at time t, which fully characterize the time-dependent behavior of the Markov chain.

As we will exemplify below, if we analyze the system knowing that it starts with certainty in a given substate i while transitioning to the absorbing substate j, and that no competing

(branched) reaction pathways lead away from substate i to a different than j substate, the time derivative of the conditional probability $p_{ij}(t)$, given that the system starts in substate i, yields the first-passage time density:

$$f_i(t) = \frac{d}{dt}p_{ij}(t) \qquad (6)$$

which describes the statistical distribution of first-arrival times from substate i to the absorbing substate j.

To apply the theory above for the purpose of extracting reaction constants from experimental observables, namely τ_{VI}, τ_{IV}, τ_{VL}, and respectively $\tau_{V/L}$ event intervals (see the corresponding description in Figure S6), we proceed as follows:

(1) By monitoring the system evolution as it transitions first time from the vestibule (V) substate to either intermediary substate (I) (quantified experimentally by τ_{VI} intervals) or lumen substate (L) (quantified experimentally by τ_{VL} intervals), the rate matrix can be simplified by discarding the subsequent evolution of the system upon either event, so that:

$$Q = \begin{pmatrix} -k_{VI} - k_{VL} & k_{VI} & k_{VL} \\ 0 & 0 & 0 \\ 0 & 0 & 0 \end{pmatrix} \qquad (7)$$

Under these circumstances, $p_{VV}(t) = e^{t(-k_{VL}-k_{VI})}$ and represents the probability of finding at a particular 't' the system in the same substate V from where it initially started. It then follows that at the same particular time 't', the probability of finding the system first transitioned from its initial substate V to either I or L equals $1 - p_{VV}(t) = 1 - e^{t(-k_{VL}-k_{VI})}$, and gives the probability that the maximum time value when the escape takes place is

precisely 't'. Based upon statistical considerations, the probability density function of the time values set constructed from τ_{VI} and τ_{VL} intervals which illustrate escapes from the initial substate V to either I or L, writes:

$$pdf\ VV(t) = \frac{\partial\left(1 - e^{t(-k_{VL} - k_{VI})}\right)}{\partial t} = e^{-t(k_{VI} + k_{VL})}(k_{VI} + k_{VL}) \quad (8)$$

It follows that the theoretical average of the time values comprising τ_{VI} and τ_{VL} intervals (denoted herein by $\overline{\tau_{VV}}$) writes:

$$\overline{\tau_{VV}} = \int_0^\infty t e^{-t(k_{VI} + k_{VL})}(k_{VI} + k_{VL})dt = \frac{1}{k_{VI} + k_{VL}} \quad (9)$$

(2) By monitoring the system evolution starting in the intermediary substate (I) as it transitions for the first time to vestibule (V) (quantified experimentally by τ_{IV} intervals), the rate matrix can be simplified as:

$$Q = \begin{pmatrix} 0 & 0 & 0 \\ k_{IV} & -k_{IV} & 0 \\ 0 & 0 & 0 \end{pmatrix} \quad (10)$$

The solution of Equation (3) in terms of conditional probability transition from the I to V substate becomes:

$$p_{IV}(t) = 1 - e^{-k_{IV}t} \quad (11)$$

and respectively the first-passage time density function of the corresponding τ_{IV} intervals write:

$$f_{IV}(t) = \frac{\partial(p_{IV}(t))}{\partial t} = e^{-tk_{IV}}k_{IV} \quad (12)$$

It then follows that average time values ($\overline{\tau_{IV}}$) obtained by quantifying τ_{IV} intervals is:

$$\overline{\tau_{IV}} = \int_0^{\infty} t e^{-tk_{IV}} k_{IV} dt = \frac{1}{k_{IV}} \qquad (13)$$

(3) Equations (9) and (13) are insufficient to form a consistent system from which the three unknown parameters — k_{VI}, k_{VL} and k_{IV} — can be uniquely determined. To get around this, we turn to the general Scheme (1) described by Equation (3) with the rate matrix (Q) embodied by Equation (5), and calculate the conditional probability for the (first) transition from the V to L substate ($p_{VL}(t)$):

$$p_{VL}(t) = 1 - e^{-\frac{1}{2}t(k_{IV}+k_{LV}+k_{VI})} Cosh[\tfrac{1}{2}t\sqrt{k_{IV}^2 + 2k_{IV}(-k_{LV}+k_{VI}) + (k_{LV}+k_{VI})^2}] - \frac{e^{-\frac{1}{2}t(k_{IV}+k_{LV}+k_{VI})} Sinh[\frac{1}{2}t\sqrt{-4k_{IV}k_{LV}+(k_{IV}+k_{LV}+k_{VI})^2}](k_{IV}-k_{LV}+k_{VI})}{\sqrt{-4k_{IV}k_{LV}+(k_{IV}+k_{LV}+k_{VI})^2}} \qquad (14)$$

As substate (L) is absorbing, the time derivative of Equation (14) represents the first-passage time density function of the corresponding $\tau_{V/L}$ intervals ($f_{VL}(t)$), which describe the times needed for the system initially found in the V substate to reach the L substate for the first time directly, or having transitioned first multiple times and reversibly to and from the I substate (see Fig. 4, I, b, c and Fig. S3, I).

$$f_{VL}(t) = \frac{\partial(p_{VL}(t))}{\partial t} = e^{-\frac{1}{2}t(k_{IV}+k_{LV}+k_{VI})} k_{LV} \left(Cosh[\tfrac{1}{2}t\sqrt{k_{IV}^2 + 2k_{IV}(-k_{LV}+k_{VI}) + (k_{LV}+k_{VI})^2}] - \frac{Sinh[\frac{1}{2}t\sqrt{k_{IV}^2+2k_{IV}(-k_{LV}+k_{VI})+(k_{LV}+k_{VI})^2}](-k_{IV}+k_{LV}}{\sqrt{k_{IV}^2+2k_{IV}(-k_{LV}+k_{VI})+(k_{LV}+k_{VI})^2}} \right. \qquad (15)$$

As above, it follows that average time values ($\overline{\tau_{V/L}}$) obtained by quantifying $\tau_{V/L}$ intervals is given by:

$$\overline{\tau_{V/L}} = \int_0^{\infty} t f_{VL}(t) dt = \frac{k_{IV}+k_{VI}}{k_{IV}k_{LV}} \qquad (16)$$

By combining equations 9, 13 and 16, one arrives at a consistent system from which the unknown parameters — $k_{\mathrm{VI}}, k_{\mathrm{VL}}$ and k_{IV} — can be uniquely determined, based on the experimentally available parameters $\overline{\tau_{VV}}$, $\overline{\tau_{IV}}$ and $\overline{\tau_{V/L}}$, respectively (Table S1)

Table S1. pH-dependent, descriptive kinetics of the captured c-MycC52 i-motif inside α-HL in terms of average values of residence and first passage times ascribed to Scheme 1.

	$\overline{\tau_{VV}}$ (s)	$\overline{\tau_{IV}}$ (s)	$\overline{\tau_{V/L}}$ (s)
**pH_{cis} = 4.76**, ΔV = +120 mV	0.004	0.002	0.5
**pH_{cis} = 5.65**, ΔV = +120 mV	0.02	0.005	0.1
**pH_{cis} = 6.53**, ΔV = +120 mV	0.02	0.03	0.8

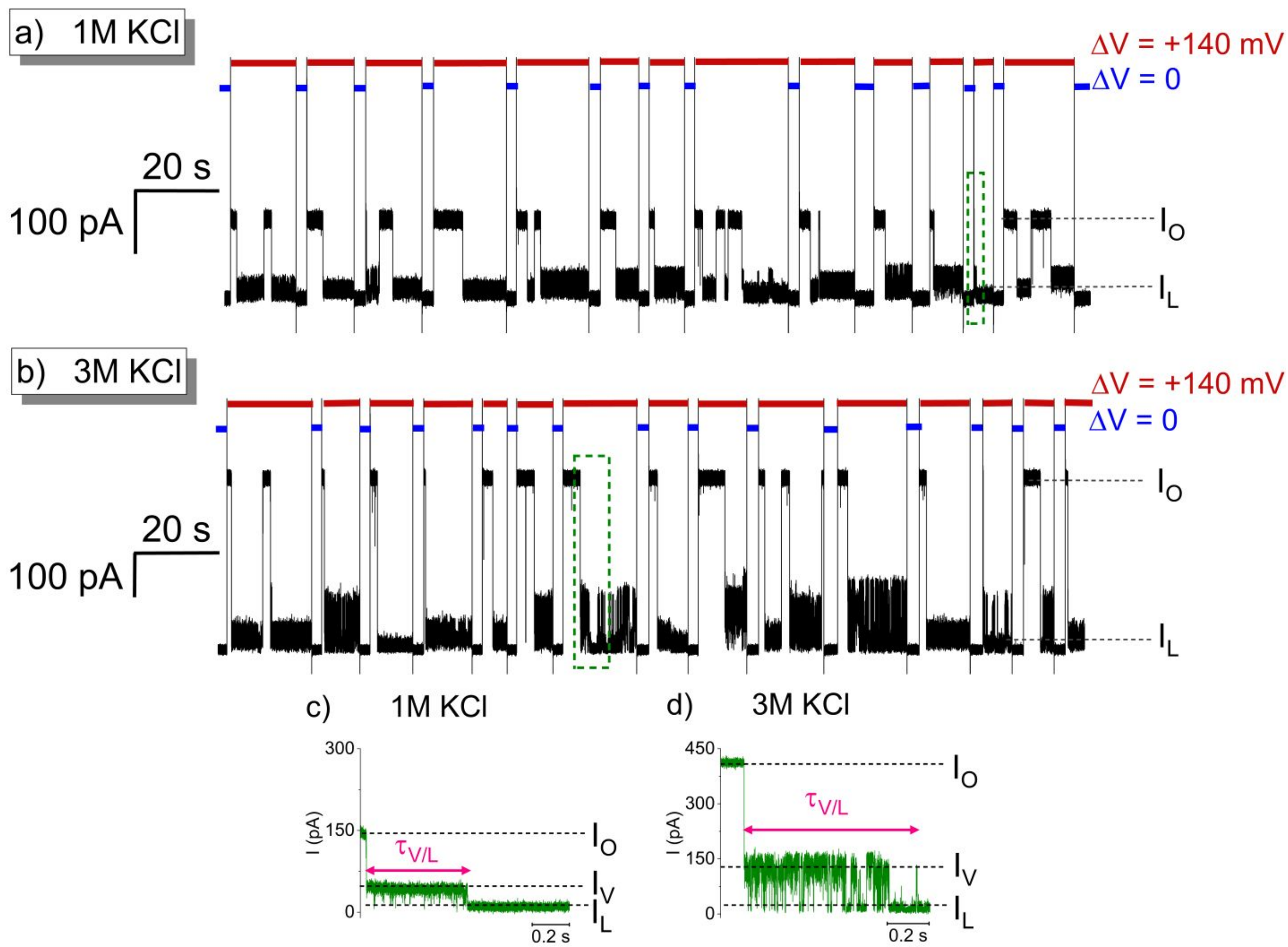

Figure S7**. Effect of ionic strength on vestibule-capture dynamics of the c-MycC52 i-motif.** Representative single-channel traces of cis-added 1 μM c-MycC52 DNA captured by a positively biased α-HL nanopore (ΔV = +140 mV) in 1 M KCl (a, c) and 3 M KCl (b, d), both buffered at the start of the experiment with 10 mM MES (pH_{cis} = 5.33, pH_{trans} = 7.14). The transmembrane potential was alternated between ΔV = +140 mV (red bars) and 0 mV (blue bars), with blockade events observed only during the voltage-on intervals. Dashed lines indicate I_O and I_L. Zoomed-in excerpts (c, d; green) resolve the I_O, I_V and I_L levels, and $\tau_{V/L}$ has a similar meaning as described

previously. At 3 M KCl, $\tau_{V/L}$ values are markedly longer than at 1 M KCl, indicating that higher ionic strength stabilizes the vestibule-entrapped state and slows the V → L transition.

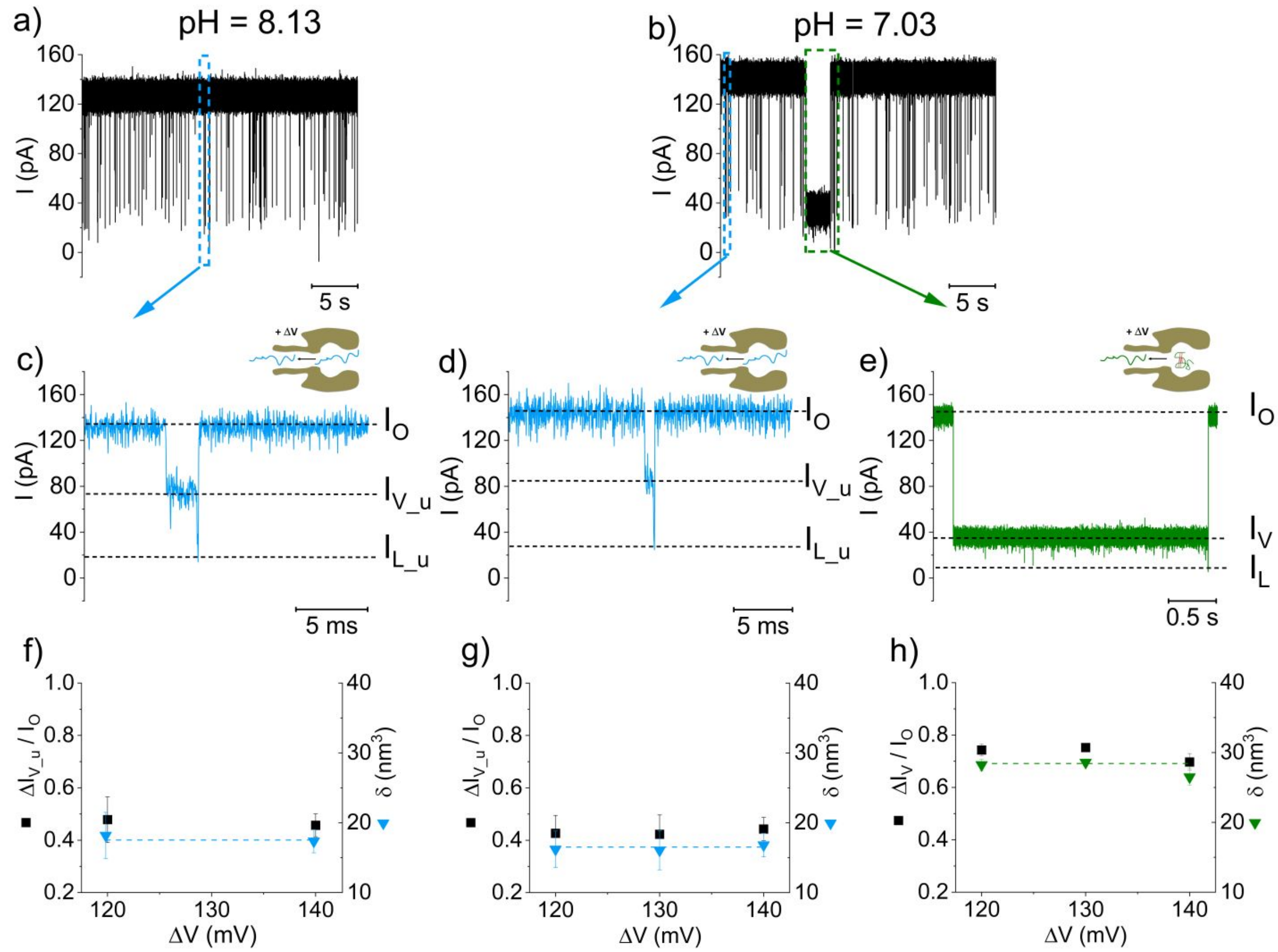

Figure S8. **α-HL-based volumetric discrimination of folded and unfolded c-MycC52 fragment at near-neutral pH in 1 M KCl**. Representative single-channel traces of cis-added c-MycC52 DNA [1 μM] captured by a positively biased (ΔV = +140 mV) α-HL in symmetrical 1 M KCl, 10 mM HEPES, at pH = 8.13 (a) and pH 7.03 (b). At pH = 8.13, only unfolded DNA species are observed, whereas at pH = 7.03 both unfolded and folded i-motif populations coexist, highlighted by the zoomed-in excerpts resolving current levels (I_O, I_{V_u} and I_{L_u}) corresponding to

(c) unfolded DNA at pH = 8.13 (blue), (d) unfolded DNA at pH = 7.03 (blue) and respectively current levels (I_O, I_V and I_L) corresponding (e) folded i-motif at pH = 7.03 (green). Accompanying schematics illustrate the translocation of unfolded DNA through α-HL (c, d) and the capture of a folded i-motif within the nanopore vestibule (e). Panels f and g show the voltage dependence of the relative blockade amplitude, $\Delta I_{V\_u} / I_O$ for unfolded DNA and panel h displays the voltage dependence of the relative blockade amplitude, $\Delta I_V / I_O$ for the folded i-motif (black squares, left axis), together with the corresponding molecular volume, δ (triangles, right axis). The horizontal dashed line represents a zero-slope linear fit (slope constrained to zero), whose constant value yields δ. The resulting molecular volumes (mean ± SEM) are $\delta = 17.5 \pm 0.3$ nm³ for unfolded DNA at pH = 8.13 (f), $\delta = 16.5 \pm 0.2$ nm³ for unfolded DNA at pH = 7.03 (g), and $\delta = 28.4 \pm 0.3$ nm³ for the folded i-motif at pH = 7.03 (h).

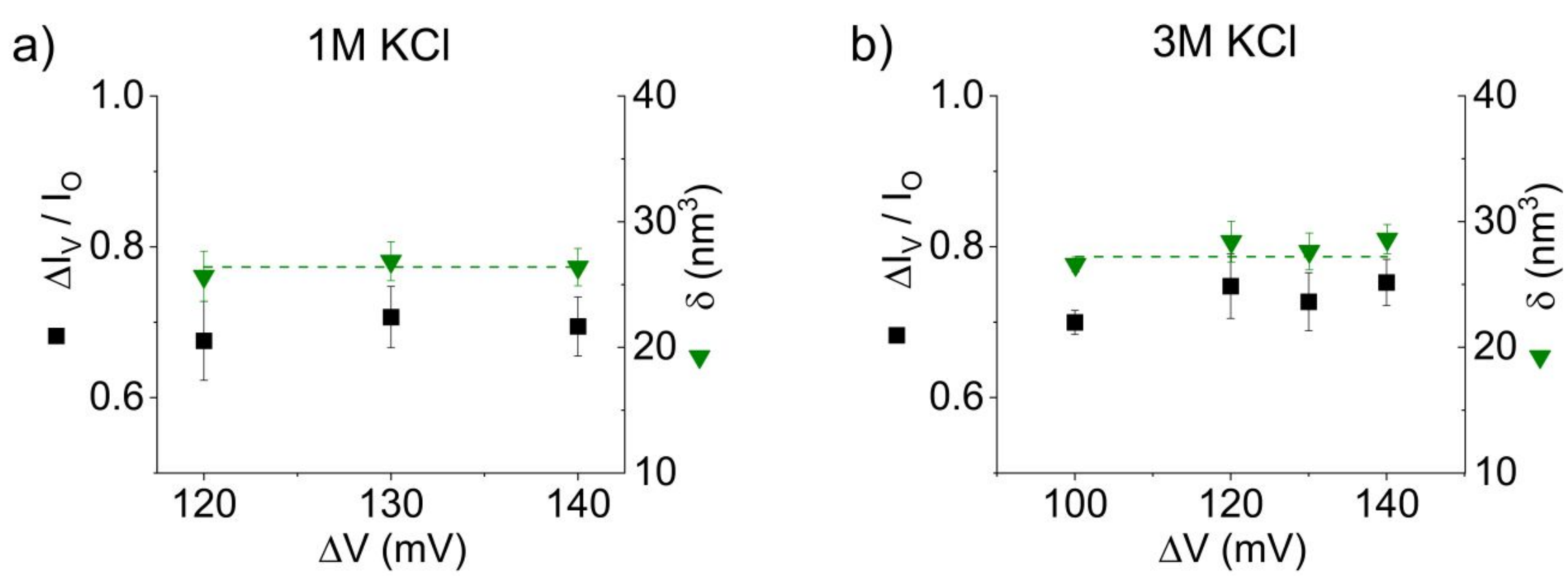

Figure S9. **Effect of ionic strength on the molecular volume of the vestibule-entrapped c-MycC52 i-motif.** Voltage dependence of the relative vestibule blockade, $\Delta I_V / I_O$ (black squares, left axis), and the corresponding derived molecular volume, δ (green triangles, right axis), for cis-added c-MycC52 [1 μM] captured by a positively biased α-HL nanopore in 1 M KCl (a) and 3 M

KCl (b), both buffered at the beginning of the experiment with 10 mM MES at pH_{cis} = 5.33 and pH_{trans} = 7.14. The horizontal dashed line represents a zero-slope linear fit (slope constrained to zero), whose constant value yields the molecular volume δ. The resulting volumes (mean ± SEM) are $\delta = 26.4 \pm 0.3$ nm³ for 1 M KCl (a) and $\delta = 27.2 \pm 0.5$ nm³ for 3 M KCl (b). The close agreement between these values indicates that ionic strength exerts no significant effect on the estimated molecular volume of the vestibule-entrapped i-motif.

REFERENCES

(1) Lakshminarayanaiah, N. Equations of Membrane Biophysics, Academic Pres, Inc, 1984

(2) Colquhoun, D.; Hawkes, A. G. The Principles of the Stochastic Interpretation of Ion-Channel Mechanisms. In *Single-Channel Recording*; Plenum Press: New York, NY, 1995; pp 397–482.
